# Timescales for Prebiotic Photochemistry Under Realistic Surface Ultraviolet Conditions

**DOI:** 10.1089/ast.2020.2335

**Published:** 2021-09-16

**Authors:** Paul B. Rimmer, Samantha J. Thompson, Jianfeng Xu, David A. Russell, Nicholas J. Green, Dougal J. Ritson, John D. Sutherland, Didier P. Queloz

**Affiliations:** ^1^ Department of Earth Sciences, University of Cambridge, Cambridge, United Kingdom.; ^2^ Cavendish Laboratory, University of Cambridge, Cambridge, United Kingdom.; ^3^ MRC Laboratory of Molecular Biology, Cambridge, United Kingdom.

**Keywords:** Photochemistry, Young Sun, Early Earth, Prebiotic chemistry, Origins of life

## Abstract

Ultraviolet (UV) light has long been invoked as a source of energy for prebiotic chemical synthesis, but experimental support does not involve sources of UV light that look like the young Sun. Here we experimentally investigate whether the UV flux available on the surface of early Earth, given a favorable atmosphere, can facilitate a variety of prebiotic chemical syntheses. We construct a solar simulator for the UV light of the faint young Sun on the surface of early Earth, called StarLab. We then attempt a series of reactions testing different aspects of a prebiotic chemical scenario involving hydrogen cyanide (HCN), sulfites, and sulfides under the UV light of StarLab, including hypophosphite oxidation by UV light and hydrogen sulfide, photoreduction of HCN with bisulfite, the photoanomerization of α-thiocytidine, the production of a chemical precursor of a potentially prebiotic activating agent (nitroprusside), the photoreduction of thioanhydrouridine and thioanhydroadenosine, and the oxidation of ethanol (EtOH) by photochemically generated hydroxyl radicals. We compare the output of StarLab to the light of the faint young Sun to constrain the timescales over which these reactions would occur on the surface of early Earth. We predict that hypophosphite oxidation, HCN reduction, and photoproduction of nitroprusside would all operate on the surface of early Earth in a matter of days to weeks. The photoanomerization of α-thiocytidine would take months to complete, and the production of oxidation products from hydroxyl radicals would take years. The photoreduction of thioanhydrouridine with hydrogen sulfide did not succeed even after a long period of irradiation, providing a lower limit on the timescale of several years. The photoreduction of thioanhydroadenosine with bisulfite produced 2′-deoxyriboadenosine (dA) on the timescale of days. This suggests the plausibility of the photoproduction of purine deoxyribonucleotides, such as the photoproduction of simple sugars, proceeds more efficiently in the presence of bisulfite.

## 1. Introduction

The starting points for origins of life research are diverse and researchers do not agree about the best place to begin. Some start with biology and investigate how extant metabolic pathways may have come to fruition, concentrating on bacterial and archaeal life that most closely resembles the last universal common ancestor. Others start with chemistry and find synthetic reactions and pathways that result in greater chemical complexity that synthesize the building blocks of the macromolecules that constitute life, such as DNA, RNA, proteins, and lipids. Yet others start with geology or exogeology, considering the mineral surfaces, volcanic degassing, atmospheric conditions, astrochemistry, and cometary chemistry and delivery of these building blocks.

Here we explore a particular scenario that started with chemical synthesis and then fruitfully underwent refinements using inputs from biochemistry and geochemistry (Sasselov *et al.*, 2020). Starting with hydrogen cyanide (HCN), hydrogen sulfide, cyanamide, cyanoacetylene, phosphates (or reduced phosphorus species, see Ritson *et al.*, 2020) in liquid water and exposing these mixtures to ultraviolet (UV) light and combining them in particular sequences result in high and selective yields of simple sugars (Ritson and Sutherland, 2012; Xu *et al.*, 2018), pyrimidine ribonucleotides (Powner *et al.*, 2009), purine deoxyribonucleosides (Xu *et al.*, 2020), other purines (Stairs *et al.*, 2017), several amino acids and phospholipid precursors (Patel *et al.*, 2015), and mechanisms for nonenzymatic activation of these (Mariani *et al.*, 2018b; Bonfio *et al.*, 2019; Liu *et al.*, 2020), or pathways to form oligomers *ab initio* (Canavelli *et al.*, 2019). Chemical pathways, especially at the start of prebiotic synthesis, must be selective and result in reasonable yields, or else the products have little chance to interact with each other to produce the macromolecules that constitute life (Sutherland, 2015; Rimmer *et al.*, 2018). This scenario therefore provides a promising chemical starting point for origins of life research (Sutherland, 2017).

The chemical starting point for this scenario has intersected multiple times with geological starting points. Hydrogen sulfide is difficult to concentrate in solution from volcanic degassing without also building up concentrations of bisulfite from dissolution of sulfur dioxide (Ranjan *et al.*, 2018, see also Gaillard and Scaillet, 2014). In closed lake environments, Ranjan *et al.* (2018) found that sulfites could have plausibly achieved 1 *M* concentrations on early Earth. The photochemistry works with bisulfite as well as hydrogen sulfide (Xu *et al.*, 2018). Bisulfite serves better for this prebiotic photochemical reduction than cyanocuprates (Todd *et al.*, 2018) or hydrogen sulfide (Rimmer *et al.*, 2018), because UV photodetachment of bisulfite electrons has a much larger cross section than the UV photodetachment of electrons from cyanocuprates or hydrogen sulfide.

These intersections do not leave this scenario unchallenged. Nonenzymatic activation of nucleotides, lipids, and amino acids can be accomplished through the intermediacy of imidazolides, which can be derived from methyl isocyanide and imidazoles (Mariani *et al.*, 2018b; Bonfio *et al.*, 2019; Liu *et al.*, 2020). Methyl isocyanide can be formed by aqueous nitroprusside chemistry, and HCN, ferrous iron, and nitrite (NO_2_^−^) react to form nitroprusside in water under UV light (Mariani *et al.*, 2018b). Fixed oxidized nitrogen on early Earth would mostly have been in the form of nitrates, whereas nitrites would likely have been less abundant on early Earth (Ranjan *et al.*, 2019). Another problem for this scenario is that, as we show, the <250 nm wavelength end of the near ultraviolet (NUV, 200–300 nm) spectrum can photodetach electrons from HO^−^, producing hydroxyl radicals that may destroy many of the photochemical products in this scenario.

This chemical scenario is directly connected to starlight, which provides a further intersection between prebiotic chemistry and astronomy. This intersection allows us to predict the planets on which life is most likely to originate via this photochemical scenario (Rimmer *et al.*, 2018). We can use this criterion to delineate an “abiogenesis zone,” outside of which the star is not bright enough in UV light to permit the photochemical scenario. This zone provides criteria informed by prebiotic chemistry for exoplanet candidate selection and ranking in the search for extrasolar biosignatures. In addition, this zone provides a test for the scenario: if life is found outside the abiogenesis zone, then there must at least be some other plausible way for life to originate. This chemical scenario also provides predictions for prebiotic chemistry on the martian surface, relevant for future missions (Sasselov *et al.*, 2020). Starlight and stellar activity affect the chemistry in other ways, by generating atmospheric HCN, relevant for prebiotic chemistry, and N_2_O, which may affect the climate (Airapetian *et al.*, 2016). The changing gas-phase chemistry will affect the average pH of open bodies of water (Krissansen-Totton *et al.*, 2018), although local lakes, ponds, and streams will plausibly have a wide range of pH, from highly acidic to alkaline (Cousins *et al.*, 2013; Toner and Catling, 2019; Kadoya *et al.*, 2020). It is also plausible that the pH will depend on the location in the body of water and will change over time (Cousins *et al.*, 2013; Mariani *et al.*, 2018a; Rimmer and Shorttle, 2019).

We consider an atmosphere favorable for UV transmission to the surface of early Earth, where photons impinge on a shallow aqueous surface environment with sufficient concentrations of cyanide, bisulfite, phosphate, or precursor reduced phosphorus, and measure the amount of time required to build up the chemical products with high (>50%) steady-state yields. To investigate these timescales, we need to simulate the young Sun's UV output within the laboratory.

All of the experiments that support, test, or challenge this scenario have been performed by using a discrete light source with most of its UV emission at a single wavelength. Most of the published experiments made use of sixteen low-pressure Hg lamps that emit in the UV almost exclusively at 254 nm. Some recent novel experiments, such as those of Roberts *et al.* (2018) and Todd *et al.* (2018, 2019), use a tunable light source and explore multiple wavelengths. None of these light sources is realistic. The Sun, like any star, is a broadband light source with a wavelength range spanning from <0.1 nm to more than 100 μm.

Habitable planets require atmospheres, and plausible abiotic atmospheres will block light <200 nm (Ranjan and Sasselov, 2016), and light at wavelengths greater than 400 nm will not typically contribute to the types of photochemistry invoked in prebiotic synthesis (*e.g*., see: Sanchez and Orgel, 1970; Ritson and Sutherland, 2012; Xu *et al.*, 2018). This leaves a broad wavelength range for photochemistry, and photochemistry is inherently nonlinear. The chemistry that is produced by a 254 nm light source and a 365 nm light source cannot be added together to determine what chemistry will occur when both are present. Prebiotic chemical scenarios that use starlight need to be investigated by using a Sun-like light source. We provide such an investigation here, and find that in some cases the chemistry does not work as well, in some cases it works better, and in some cases it seems not to work at all.

In this article, we present StarLab, an experimental stellar simulator set up to simulate the light of the Sun as it was roughly 4 billion years ago. In Section 2, we provide the schematic for the experiment and compare its output to the output of young Sun analogues. In Section 3, we discuss a group of experiments that are key for different aspects of UV-driven prebiotic chemistry, for which we use StarLab. We also run these experiments under the more “classic” illumination source of a mercury lamp reactor (the Rayonet RPR-200) to demonstrate the differences in outcome between using a more realistic broadband source such as StarLab and narrow-band sources that emit primarily at wavelengths of 254 or 365 nm (depending on the specific bulb used). We show the results of these experiments. In Section 4, we discuss how these experiments can be directly compared with an early Earth environment to constrain whether the light of the young Sun was sufficient for these reactions on early Earth. We summarize the article in Section 5.

## 2. Simulating the Young Sun

Here we discuss the design of the UV source we use in all the following prebiotic chemical experiments. In simulating the young Sun, first we discuss the design of the experimental apparatus (Section 2.1) and then discuss the plausible UV spectrum of the young Sun and compare this spectrum with the apparatus (Section 2.2).

### 2.1. StarLab: the apparatus

The top-level requirements for StabLab are as follows:

(1) To provide a broadband illumination that approximates the spectral irradiance profile of the Sun on the surface of the early Earth (preozone layer).
(2) To provide a means to deliver that illumination into a 10-mm-sided quartz cuvette sample holder, minimizing loss of photons until it arrives at the sample.
(3) To ensure that the sample does not overheat during the course of the experiment—for our purposes we defined this to be *T* < 40°C over the length of the experiment, which can range from 30 min to several days.
(4) To have a means of measuring the spectral irradiance received by the sample and to be able to monitor the change in spectral irradiance after passing through the sample.

The ozone layer formed in Earth's atmosphere sometime after the Great Oxygenation Event about 2.3 Ga (Luo *et al.*, 2016), while life on Earth is believed to have arisen around 4 billion years ago. The ozone layer blocks radiation in the 200–310 nm wavelength range. To replicate the illumination on the surface of early Earth, around the time that life was first forming, the illumination source must provide UV photons down to 200 nm. This top-level requirement, derived from Requirement (1), determines the choice of lamps available for the experiment and the types of optical materials that can be used.

The lamps available that provide broadband illumination in the 200–400 nm region are as follows:

- Xenon arc lamp, usable wavelength range: 200–2500 nm.
- Deuterium arc lamp, usable wavelength range: approximately 160–400 nm.
- Laser-driven light source, wavelength range 170–2100 nm.

Modifications to this wavelength range are dependent on the exact lamp system configuration. “Ozone-free” versions of most lamps are available, which usually block wavelengths below 260 nm (thus preventing the conversion of O_2_ to O_3_, which is hazardous in laboratories that cannot provide sufficient ventilation). Other coatings can be available for the lamps to tune the lower cutoff wavelength and fiber-coupled lamps generally limit the lower wavelength cutoff to ∼190 nm.

For the first version of our StarLab experimental configuration, we chose a 75 W Xenon arc lamp in the PowerArc range manufactured by Horiba. The lamp housing for this model uses an ellipsoidal reflector and so is very efficient at collecting and focusing most of the light emitted by the Xe bulb at a single focus (as a point of comparison, the more traditional vertical lamp housings, at one of their foci, would contain five to six times less flux for the same wattage lamp).

**FIG. 1.**
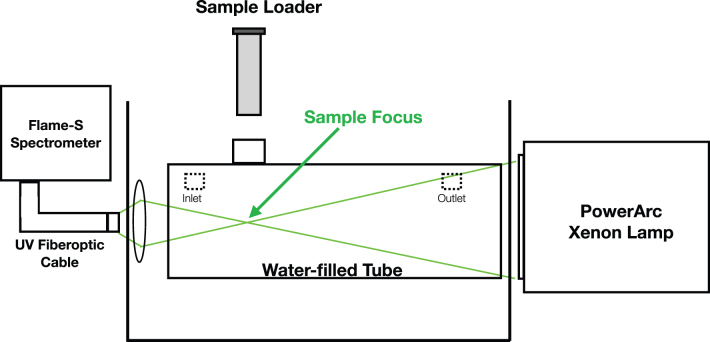
Schematic of StarLab, the stellar simulator for the young Sun. The focal distance from the aperture of the Xe lamp to the sample is 150 mm, which contains an optical depth of water in the quartz attenuator (water-filled tube) of 130 mm. The spectrometer fiber can be placed at the sample focus (via the sample loader) to measure the flux received by the sample before sample loading, or at the position shown in the drawing to monitor changes while a sample is being illuminated. Color images are available online.

**FIG. 2.**
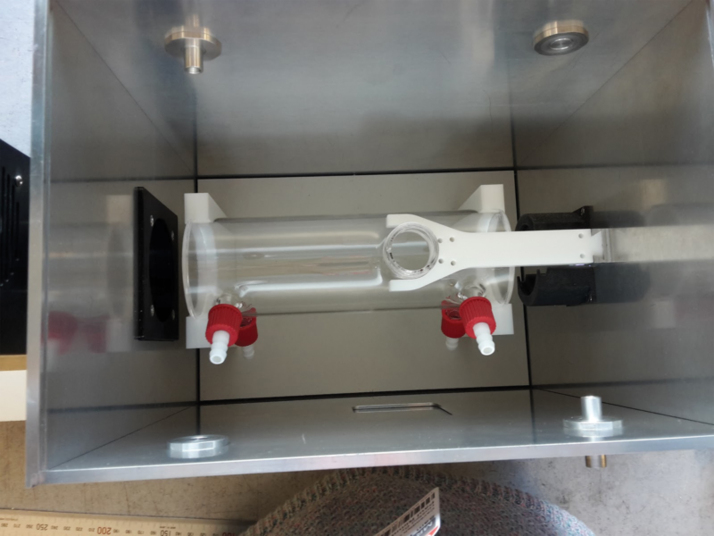
Photograph of the quartz attenuator tube inside its mounting box. The Xe arc lamp is mounted to the left, outside of the box, the black interface plate can be seen to the left inside the mounting box. The sample is loaded through the circular opening in the top of the tube so that it is positioned at the focus of the Xe lamp. Color images are available online.

One feature of the spectral irradiance of a Xe lamp that needs consideration is the high-emission region from ∼750 to 1100 nm. This high-level emission is not present in the early solar spectrum and additionally brings problems into the experimental setup due to strong heating effects. Without cooling of the sample or a prefiltering of the Xe spectrum, the sample to be illuminated by our simulated starlight would rapidly boil. To meet Requirement (3), we devised an apparatus that can provide both the functions of filtering the unwanted spectral regions and allow for the possibility of cooling the sample if required. The schematic of the StarLab apparatus is shown in Fig. 1, and a picture of the experiment is shown in Fig. 2. The setup is based around a piece of customized glassware—the glass in this case is GE 214, a type of fused quartz that has a high transmittance over our required wavelength range. This custom glassware, which we call the quartz attenuator, is filled with water and is designed to hold a sample cuvette at the focus of the Xe arc lamp. The water acts like an optical filter, preferentially absorbing the emissions from the Xe arc lamp in the near infrared and so preventing the sample from overheating. If required, the quartz attenuator can be connected to a circulating water supply so that the temperature of the water is maintained at a given value.

**FIG. 3.**
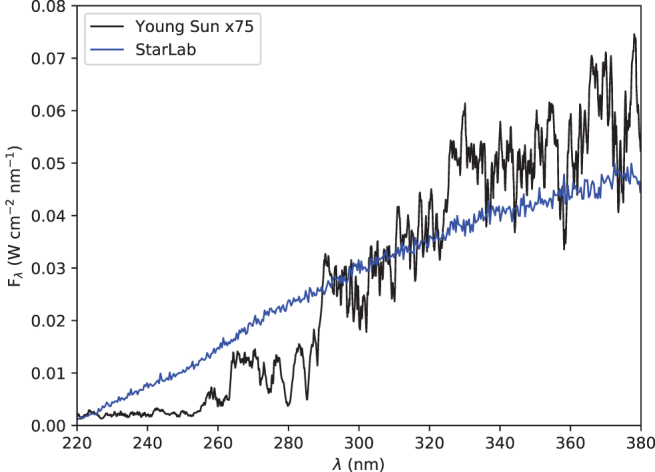
Spectral irradiance [W/(cm^2^·nm)], versus wavelength (nm), for StarLab at the location of the sample (blue), compared with 75* ×* the spectral irradiance 1 AU from the young Sun as observed above the atmosphere (black). Color images are available online.

**FIG. 4.**
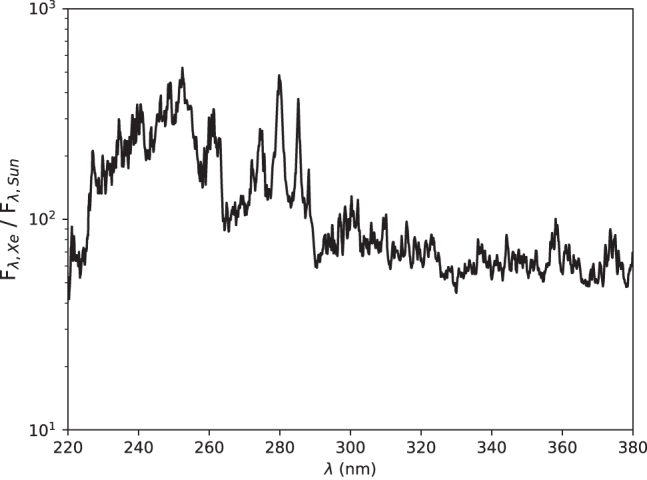
Ratio of the spectral irradiance of StarLab over the spectral irradiance 1 AU from the young Sun as observed above the atmosphere, as a function of wavelength (nm).

**FIG. 5.**
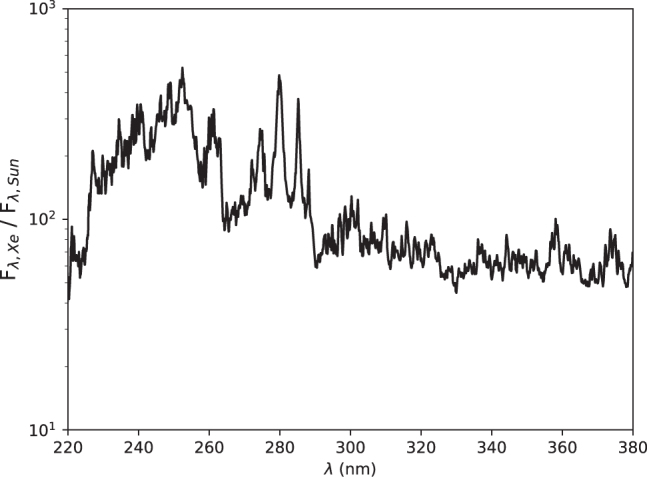
Ratio of the spectral irradiance of StarLab over the spectral irradiance at the surface of early Earth, as a function of wavelength (nm), assuming a 0.3 bar atmosphere with 33% N_2_, 33% CO, and 33% CO_2_ with water at vapor pressure.

Figure 3 shows the spectral irradiance of StarLab in the wavelength region of interest, as measured at the sample position (through the water-filled quartz attenuator), and Figs. 4 and 5 show the ratio of StarLab to the young Sun, above the atmosphere and at the surface of early Earth, respectively.

For the fourth requirement (see list above), we used an Ocean Optics (now Ocean Insight) Flame UV-VIS (visible) Spectrometer to measure the spectrum of light received by the sample at the lamp focus and to monitor any changes in the spectrum during the course of an experiment (Fig. 1)—that is, from absorption changes due to chemical evolution in the prebiotic sample. The measurement range of the Flame spectrometer is given to be 200–850 nm, however, on usage, we quickly realized that for spectral regions <220 nm, the measurements had extremely low signal-to-noise, which effectively made this region unusable for our purposes. For the set of experiments presented here, we essentially have no flux below 220 nm due to the absorption of those wavelengths by nonpure water. This is a realistic scenario for aqueous chemistry on early Earth—the scenario itself requires several chemical species to be dissolved in the “prebiotic pond-water,” which rapidly attenuates the transmission of the shorter wavelengths of UV light (see Quickenden and Irvin, 1980 and Higashi and Ozaki, 2004 for examples of UV absorption properties of different purities of water). Using the data from the work of Higashi and Ozaki (2004), we calculated the expected attenuation of a 130 mm water depth (the column depth of water in StarLab)—most of the mineral waters in that sample (similar to the Cambridge tap water used in the StarLab quartz attenuator) show a 0–0.3% throughput at 220 nm. This demonstrates that realistic water sources are very effective blockers of UV light below 220 nm. In addition, we note that for waters containing ferrocyanide we would expect the UV attenuation to be significantly higher (Chakrabarti and Roberts, 2008). So that in a postimpact pond on early Earth, assuming a few mmol of ferrocyanide, even for very shallow optical depths, almost negligible amounts of UV light <220 nm would penetrate.

Using the experimental setup shown in Fig. 1 with tap water in the quartz attenuator, we have thus considered a realistic flux for most of a body of water on early Earth, except for at the actual surface of the water, where shorter wavelength chemistry may occur.

### 2.2. Comparing StarLab with the young Sun

The Sun is a G2V-type star and is about 4.6 billion years old (Bonanno *et al.*, 2002). It exhibits a typical activity level of stars of similar age and mass (Mittag *et al.*, 2016). Using the Yonsei-Yale evolutionary track (Kim *et al.*, 2002; Yi *et al.*, 2003), its effective temperature has remained nearly constant with age, and its radius has increased with age. This means that in the past its radius was smaller. The Sun <3.5 Ga was 75% the size of the Sun today and this is the reason for the faint young Sun paradox (Sagan and Mullen, 1972). The Sun can be compared with other young solar metallicity stars that fit this evolutionary track, and the spectra of these stars can be measured.

These spectra are a proxy of the spectrum of the Sun at that age. The star *κ*1-Ceti is a star with solar metallicity and has an age of 300–400 My and matches the Yonsei-Yale evolutionary track for the Sun when it was 300–400 My old, and so, *κ*1-Ceti is an analog young Sun, and its UV spectrum is used as a proxy of the UV spectrum of the young Sun (Ribas *et al.*, 2005, 2010). Claire *et al.* (2012) use *κ*1-Ceti, along with EK Dra, *π*1-Uma, *χ*1-Ori, *β* Com, and *β* Hyi, to construct an evolutionary model of the Sun's UV spectrum from 4.5 Ga to the present. Since *κ*1-Ceti is approximately the age of the Sun 4.0 Ga, and since this seems consistent with the time life approximately originated on Earth, we compare the spectrum of our lamp with the spectrum of *κ*1-Ceti, which we hereafter call the young Sun or young solar spectrum.

Early Earth must have had an atmosphere to support liquid water, although this atmosphere may have been more tenuous 4.0 Ga than today (Som *et al.*, 2016; Avice *et al.*, 2017; Rimmer *et al.*, 2019; Lehmer *et al.*, 2020; Payne *et al.*, 2020). The light of the young Sun must traverse this atmosphere to reach the surface and the atmosphere will block some UV light. For this work, we assume a 0.3 bar atmosphere composed of 33% N_2_, 33% CO, and 33% CO_2_ with water at vapor pressure, the same as the >3.5 Ga atmosphere Rimmer *et al.* (2019) used to explain the observed micrometeorite oxidation from the work of Tomkins *et al.* (2016), and consistent with other early Earth model atmospheres invoked to explain the same data (Lehmer *et al.*, 2020; Payne *et al.*, 2020). We solve for the surface UV flux using the model of Rimmer & Helling (2016) and Rimmer and Rugheimer (2019) with the UV absorption and scattering cross sections from PHIDRATES (Huebner *et al.*, 1992; Huebner and Mukherjee, 2015) for most species, with updated cross sections for CO_2_ (Ityaksov *et al.*, 2008), HCHO (Chen and Zhu, 2001), H_2_O (Ranjan *et al.*, 2020), and CH_4_ (Burkholder *et al.*, 2020). Most of these cross sections were obtained from the Mainz database (Keller-Rudek *et al.*, 2013).

## 3. UV-Driven Prebiotic Chemistry: Methods and Results

Here we discuss the series of experiments we performed to test whether UV light is prebiotic in the context of the prebiotic chemical scenario discussed in Section 1. We answer this question in terms of the timescale of reaction, by which we here mean a photochemical “timescale,” here defined as the time it takes to achieve a 50% yield in a given reaction. This timescale is relevant for prebiotic plausibility of sequences of reactions (see Rimmer *et al.*, 2018, their figure 3). Although StarLab has the same spectral shape as the faint young Sun between 200 and 400 nm, it does not have the same intensity as that expected on the surface of early Earth, and so, the timescale in the laboratory does not directly represent but predicts the timescale on early Earth.

The rate constant at which a *pure* photochemical reaction takes a reactant to product is called the photoproduction rate, $k_{\nu }$ (s^−1^), and takes place in a unit volume, depends on the flux of photons of a particular wavelength, λ (nm), entering into a unit sphere surrounding that volume, $F_{\lambda }$ [/cm^2^/(s·Å)], which is related to the spectral irradiance (which our spectrometer measures) by a factor of hc∕λ, the individual photon's energy. It also depends on the probability that a given photon of wavelength λ is absorbed by the reactant, which is represented by the absorption cross section, $\sigma_{a,\lambda }$ (cm^2^), multiplied by the probability that the product results from the absorption of that photon, called the quantum yield, the unitless quantity $\phi_{\lambda }$. We define a reaction cross section as $\sigma_{\lambda }=\sigma_{a,\lambda }\phi_{\lambda }$. The photoproduction rate is then:

(1) $$k_{\nu }=\int \sigma_{\lambda }F_{\lambda }d\lambda$$

When the photochemical process is known to be linearly dependent on concentration and that there are no other processes that provide significant competition over the same timescale as the irradiation, then the time it takes for a reaction to achieve a yield, *f*, can be related to the photoproduction rate, $k_{\nu }$ (s^−1^), by the following equation:

(2) $$k_{\nu ,exp}=\frac{1}{t_{exp}}log\left(\frac{1}{1-f}\right)$$

The advantage of reproducing a plausible UV spectrum for the young Sun is that we can then scale this experimental rate by noting that $F_{\lambda ,exp}=\alpha \left(\lambda \right)F_{\lambda ,sun}$ and so, insofar as α is constant with respect to λ:

(3) $$k_{\nu ,exp}=\int \sigma_{\lambda }F_{\lambda ,exp}d\lambda =\alpha \int \sigma_{\lambda }F_{\lambda ,sun}d\lambda$$

(4) $$=\alpha k_{\nu ,sun}$$

We can relate this to Eq. 2 and can then relate experimental time to the time it would take to complete the same reaction on the surface of early Earth, noting that the timescale on early Earth (and for any planet *i.e*., not tidally locked) will be twice as long to account for the day/night cycle. First, we define an “irradiation time,” $t_{irr}eqnoopen\left(s\right)$, which is the amount of time a sample would need to be irradiated on early Earth to match the irradiation by StarLab:

(5) $$t_{irr}=\alpha t_{exp},$$

Since the environment is only irradiated half the time on early Earth, the actual timescale for the reaction, against which the degradation timescales of the reactions must compete, is as follows:

(6) $$t_{Earth}=\frac{\alpha }{2}t_{exp}.$$

We return to this concept and explore the wavelength dependence of α in Section 4. For now, we relate the outcome of a series of experiments exemplifying different parts of the UV-driven prebiotic chemical scenario to this timescale. This timescale can be compared with the degradation timescales of the reactants, or other relevant chemical timescales, to test the plausibility of these reactions on the surface of early Earth.

We begin with a summary of the UV-driven prebiotic chemical scenario (Section 3.1), and then discuss a selection of key photochemical reactions in order. We start with the oxidation of hypophosphite with hydrogen sulfide and UV light (Section 3.2), and then proceed to the photoreduction of HCN by solvated electrons from bisulfite (Section 3.3), then the photoanomerization of α-thiouridine (Section 3.4), the attempted photoreduction of thioanhydrouridine (Section 3.5), the photoreduction of thioanhydroadenosine (Section 3.6), the photoproduction of nitroprusside (Section 3.7), and the oxidation of ethanol (EtOH) by photoproduced hydroxyl radicals (Section 3.8).

### 3.1. Overview of the scenario

**FIG. 6.**
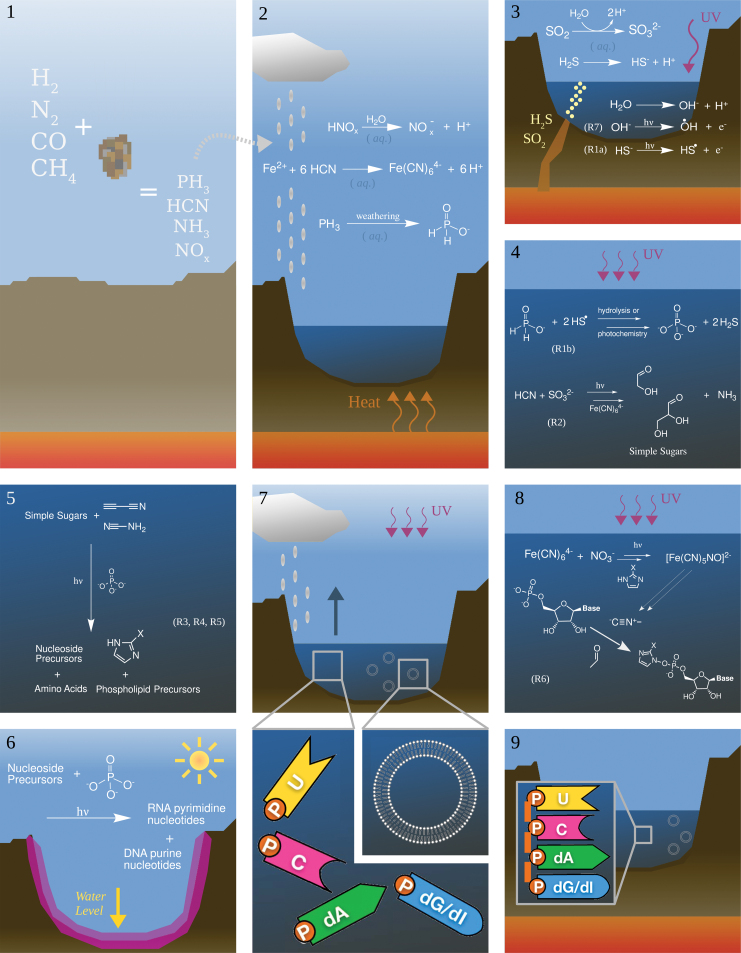
A description of the UV-driven prebiotic chemical scenario. **(1)** An impactor strikes the surface of Earth, generating significant amounts of PH_3_, HCN, NH_3_, and NO_x_. **(2)** These species rain out and form a crater lake with a variety of organometallic complexes and salts, such as ferrocyanide. **(3)** Volcanic gas bubbles into the crater lake and UV light impinges on the lake surface, generating OH (R7, Section 3.8) and HS radicals (R1a, Section 3.2). **(4, 5)** The HS radicals participate in the oxidation of PH_3_ to phosphates (R1b, Section 3.2) and the UV light generates solvated electrons from bisulfite. The solvated electrons react with the HCN and simple sugars are formed (R2, Section 3.3). These simple sugars undergo a series of reactions with cyanamide and cyanoacetylene to form nucleotide, amino acid, and phospholipid precursors (steps within this series of reactions are discussed in Sections 3.4–3.6, R3–R5). **(6)** The lake dries, allowing for phosphorylation of the nucleosides and formation of phospholipids. **(7)** Rain fills the lake with water again, and the phospholipids form into vesicles. Amino acids and precursors to RNA and DNA are also present in the solution. **(8, 9)** The NO_2_^−^ and nitrate salts react with ferrocyanide to form nitroprusside (R6, Section 3.7), precursors for methyl isocyanide, a nonenzymatic activating agent that leads to the formation of hybrid RNA/DNA oligomers and polypeptides. HCN, hydrogen cyanide*;* NH_3_, ammonia; NO_2_^−^, nitrite; PH_3_, phosphine; UV, ultraviolet. Color images are available online.

The series of reactions discussed in this article are best understood in the context of a *scenario*—a sequence of chemical reactions that takes place in a specific geochemical environment. The scenario we discuss here is shown in Fig. 6.

We begin with Earth after the single moon-sized impactor or multiple smaller impactors provided the material for the late veneer (Genda *et al.*, 2017), which according to Brasser *et al.* (2020) occurred at ∼4.48 Ga. Models of the chemical effect of this bombardment suggest that the impact produces a transient reducing atmosphere that could have persisted for a 100 million years (Zahnle *et al.*, 2020). We focus on one such surface, on a volcanic island.

Another impact, at the tail end of accretion, strikes this surface, generating a plasma shock that results in a considerable amount of HCN, reduced phosphorous species, ammonia (NH_3_), and, depending on atmospheric redox, some nitric oxide and nitrogen dioxide (NO and NO_2_). These species subsequently rain out onto an Fe(II)-rich surface, where the HCN forms ferrocyanide salts (Patel *et al.*, 2015, see their figure 2), hypophosphite is among the reduced phosphorous species (Ritson *et al.*, 2020), and the NO and NO_2_ are oxidized to form nitrites and nitrates (Ranjan *et al.*, 2019), but dominantly nitrates.

Impact craters provide ideal environments for surface hydrothermal vents (Bryant *et al.*, 2013). The pool that intersects with a hydrothermal vent has sulfur dioxide and hydrogen sulfide bubbling through, and if the pH is sufficiently high, ≳7, the sulfur dioxide reacts with the water, forming sulfite and bisulfite salts, and the hydrogen sulfide dissociates, forming sulfides (Ranjan *et al.*, 2018). This pH is plausible for surface hydrothermal systems on volcanic islands, even under CO_2_-rich atmospheres (Cousins *et al.*, 2013).

As water will self-dissociate producing hydroxide (OH^−^) anions, UV light will impinge on both OH^−^ and sulfide (HS^−^), resulting in solvated electrons in addition to OH and HS radicals. The photodetachment of an electron from HS^−^ is the first step of the reaction discussed in Section 3.2, and HS radicals can oxidize hypophosphite, resulting in the phosphate required as a chemical and pH buffer for subsequent prebiotic chemistry. The oxidation of hypophosphite with HS radicals is the second step of the reaction discussed in Section 3.2. The photodetachment of an electron from OH^−^ is the reaction discussed in Section 3.8 and OH radicals can destroy the products of prebiotic chemistry.

UV light will also impinge on the sulfite salts (SO_3_^2−^), efficiently producing solvated electrons (Rimmer *et al.*, 2018; Xu *et al.*, 2018). These solvated electrons can add to HCN, which will be reduced and subsequently hydrolyzed. Addition of excess HCN and further reduction can then form glycolaldehyde and glyceraldehyde (Xu *et al.*, 2018). The production of solvated electrons and the subsequent chemistry that results in simple sugars are the reactions discussed in Section 3.3.

Impacts will also result in cyanamide and cyanoacetylene, either directly (Patel *et al.*, 2015) or from further chemical alteration within a volcanic system (Rimmer and Shorttle, 2019). These and phosphates undergo a series of reactions with simple sugars to produce pyrimidine ribonucleotides, purine deoxyribonucleotides, amino acids, and phospholipids (*e.g*., Patel *et al.*, 2015), the building blocks of RNA, DNA, proteins, and cell membranes. Key reactions along the way to the selective, high-yield synthesis of these species involve UV light, and we examine these in Sections 3.4–3.6.

These building blocks will not react with each other unless activated. In biology, these molecules are activated and then joined together by enzymes. Fortuitously, chemical by-products of the UV-driven synthesis described above react together to activate amino acids, nucleotides, and phospholipids (Bonfio *et al.*, 2019; Liu *et al.*, 2020). This is initiated when the nitrites and nitrates, mentioned above, interact with UV light in the presence of ferrocyanide, producing nitroprusside, via the reaction discussed in Section 3.7.

Nitroprusside undergoes a further series of reactions (Mariani *et al.*, 2018b), resulting in methyl isocyanide, which, in tandem with imidazoles (by-products of reactions involving those in Sections 3.4–3.6), perform the activation and ligation. Although this system has not been tested altogether, the projected result of the scenario would be phospholipid membranes containing a DNA/RNA hybrid system, such as that discussed in the works of Bhowmik and Krishnamurthy (2019) and Xu *et al.* (2020) (see Section 3.6 for details) with nonenzymatic activating agents, coupled in some way to polypeptides (as discussed broadly in Liu *et al.*, 2020). There is much work to be done to determine the plausible next steps toward the origin of the genetic code and modern metabolism (see Wu and Sutherland, 2019).

We do not test every photochemical reaction in the network, and so, there is ample work for the future. We do test key reactions at each stage of the synthesis and activation of the building blocks of life within this UV-driven prebiotic scenario, which provides an overview of the “landscape” of prebiotic plausibility in terms of the available UV flux. In the same vein, no one need be wedded to this exact scenario, which is provided here mostly to illustrate how the chemistry can be connected. Any scenario that involves these products in an environment accessed by UV light could involve the reactions we now discuss.

### 3.2. Hypophosphite oxidation with bisulfide

We first consider the photochemical oxidation of hypophosphite (H_2_PO_2_^−^) to phosphate (PO_4_^3−^) in the presence of hydrogen sulfide.

The availability of phosphorus on early Earth, both in terms of the form the phosphorus would likely take and the amount available for prebiotic synthesis, is unknown. After planetary differentiation, the small amount of phosphorus residing in the lithosphere would have been trapped almost exclusively in the form of apatite (Nash, 1984). The near-insolubility of apatite in water resulted in the so-called phosphate problem, as pointed out many years ago by Gulick (1955), although recently a solution to this problem has been proposed for carbonate-rich environments (Toner and Catling, 2019).

Soluble phosphorus can also result from the aqueous alteration of schreibersite (Tackett *et al.*, 1966), a mineral common in iron/nickel meteorites, but this typically results in reduced forms of phosphorus such as phosphite and hypophosphite (Bryant *et al.*, 2013; Kee *et al.*, 2013). Impacts may plausibly convert large amounts of phosphorus into phosphine (Ritson *et al.*, 2020), which can then be oxidized in the atmosphere—a process that still occurs on Earth today, although in trace amounts, and presumably proceeds via phosphine oxide, hypophosphite, and phosphite, eventually leading to phosphate if oxidation is complete (Glindemann *et al.*, 2003). As Gulick (1955) also pointed out, phosphite and hypophosphite are more soluble than phosphate in the presence of divalent metal ions (in particular calcium and iron), but would not appear to be as straightforwardly useful for prebiotic chemistry given the enhanced lability of these groups when incorporated into organic molecules and the lack of ability of hypophosphite to form phosphodiesters, which would be required for an RNA or DNA analogue.

Hypophosphite can be converted to phosphate by reaction with hydrogen sulfide and UV light (Ritson *et al.*, 2020). This is the reaction we explore here.

Hypophosphorous acid 50% wt (0.055 mmol, 6 μL) was mixed into argon-degassed 10% D_2_O/90% H_2_O (6 mL), and the pH was adjusted to 7.0 by using degassed NaOH. Then NaSH.xH_2_O (0.034 mmol, 1.9 mg) was added to the mixture, and the pH was again slowly adjusted to 7.0 by using degassed HCl. This volume was divided equally into two cuvettes, one irradiated by StarLab and the other by the photoreactor RPR-200. The samples were irradiated for 1 h.

**FIG. 7.**
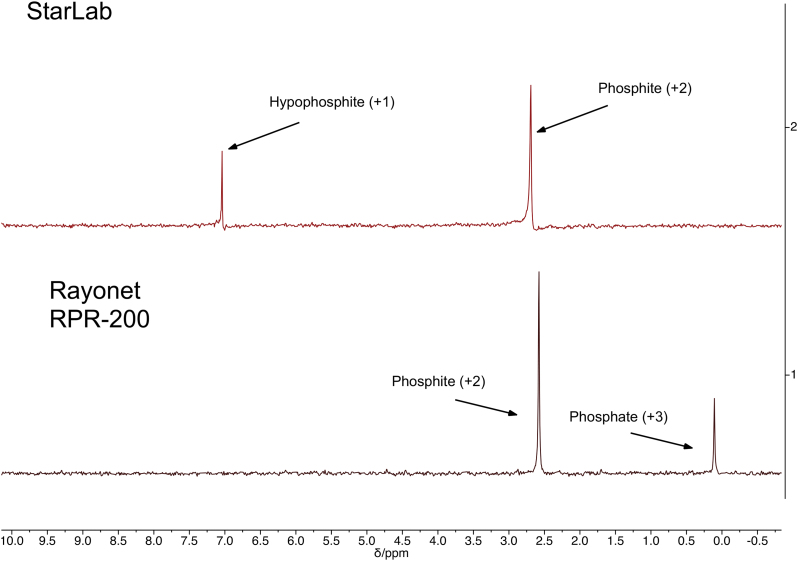
31P NMR showing the reaction of hypophosphite and HS^−^ in water irradiated for 1 h with (top) StarLab and (bottom) the RPR-200 (Rayonet). After 1 h, with StarLab, on the order of 50% of the starting material has been consumed and converted to phosphite. Under the Rayonet, all of the starting material is consumed, converted mostly to phosphite with some phosphate. Color images are available online.

As can be seen from the nuclear magnetic resonance spectrum, shown in Fig. 7, after 1 h of irradiation with StarLab, more than 50%, but not substantially more than 50%, of the hypophosphite has been oxidized to phosphite, which suggests a chemical timescale for this conversion of 1 h, suggesting that this reaction at these concentrations would reach this point after 36–60 h on prebiotic Earth. One-hour irradiation with the RPR-200 results in nearly 100% yield.

### 3.3. HCN reduction with bisulfite

The next reaction we consider is as follows:

**7:**
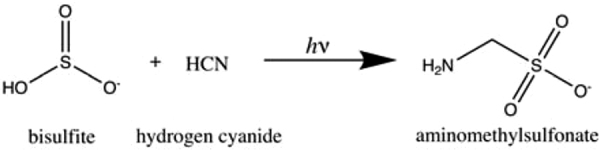


UV light generates solvated electrons from bisulfite, and these solvated electrons can reduce HCN (Xu *et al.*, 2018). This is the first step toward the synthesis of simple sugars and nitriles (Xu *et al.*, 2018), leading to nucleotides, amino acids, and phospholipid precursors (Patel *et al.*, 2015). It is also the key reaction that Rimmer *et al.* (2018) used to delineate the abiogenesis zone. Aminomethylsulfonate is the adduct of bisulfite with the first reduced product of HCN, and the concentration of aminomethylsulfonate allows us to estimate both the yield and the rate of this reaction.

For this reaction, ^13^C-labeled KCN (0.15 mmol, 10.0 mg), Na_2_SO_3_ (0.60 mmol, 76.0 mg), and NaH_2_PO_4_ (0.61 mmol, 73.0 mg) were mixed in argon-degassed H_2_O (5 mL, 10% D_2_O/90% H_2_O). The pH of the mixture was adjusted to 7.0 with 1 *M* HCl. The mixture was then divided equally into two 10 mm quartz cuvettes, one of which was irradiated at 254 nm in a photochemical chamber reactor RPR-200, while the other sample was irradiated in StarLab for the same time.

**FIG. 8.**
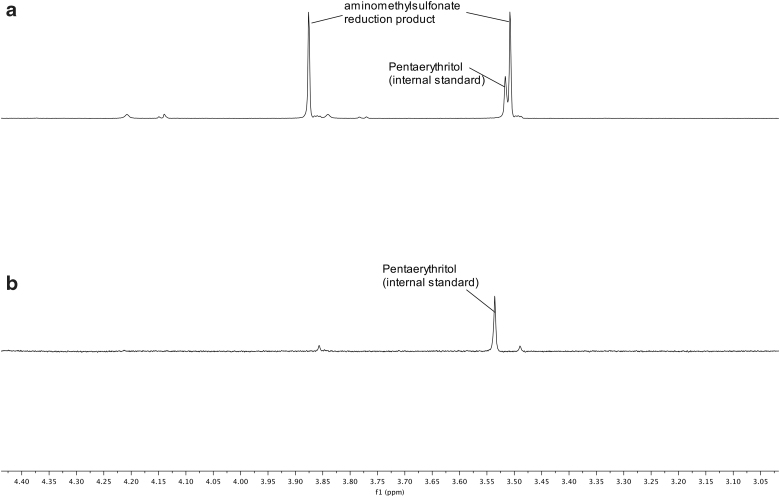
^1^H NMR spectrum of the photoreduction of HCN in the presence of bisulfite, using pentaerythritol as a standard for comparison, after 1 h. The concentration of product is calculated by integrating under the curve for the aminomethane sulfonate and comparing the value with the integral under the curve of pentaerythritol. **(a)** Using the RPR-200 reactor. The reduction product is clearly present and the area under the curve exceeds that of the pentaerythritol. **(b)** Using StarLab. The reduction product is far less than **(a)**, but still visible.

**FIG. 9.**
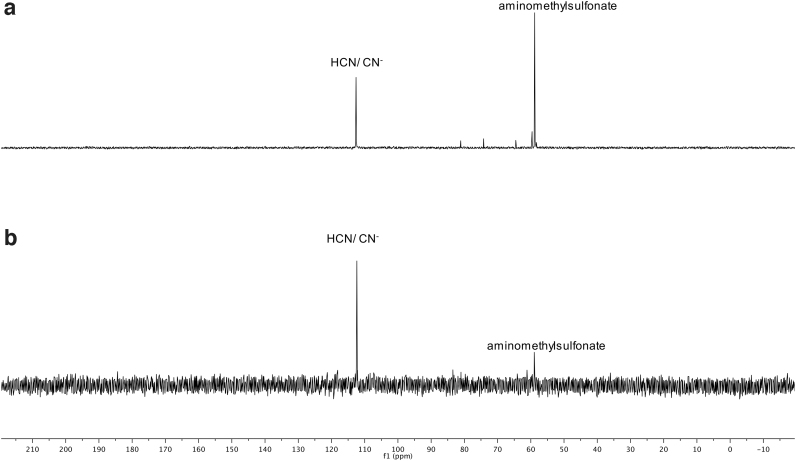
^13^C NMR spectrum of the photoreduction of HCN in the presence of bisulfite, using pentaerythritol as a standard for comparison, after 1 h, the same reaction as shown in Fig. 8. Although this ^13^C NMR spectrum is not quantitative, it does give us an approximate measure of the difference between reactant and product. **(a)** Using the RPR-200 reactor. The peak for the reactant has exceeded that for the product, but the product is still present in ^13^C NMR. Compare with Fig. 8a. **(b)** Using StarLab. The peak for the reactant is visible but less than for the product, and far less than for the RPR200 reactor. Compare with Fig. 8b.

We present the resulting ^1^H and ^13^C NMR spectra in Figs. 8 and 9. Comparing the integration of pentaerythritol (added as a reference) and aminomethylsulfonate for the StarLab signals suggests a yield of 10% and a $k_{\nu ,exp}=1.3\times 10^{-5}$ s^−1^. We can apply this estimate to Eqs. 2 and 3 and apply the measured $\sigma_{\lambda }=1.5\times 10^{-21}$ cm^2^ (Rimmer *et al.*, 2018) and the StarLab's spectral output for the sample (Figs. 3–5), to test whether the cutoff wavelength for the electron photodetachment reaction really is at 280 nm, or is at a different wavelength. We find that the cutoff wavelength is more likely at 260 nm. If the cutoff wavelength were much higher, the concentration after 1 h should be greater. This cutoff is 20 nm lower than assumed by Rimmer *et al.* (2018).

### 3.4. Photoanomerization of α-thiocytidine

The third reaction we consider is as follows:

**8:**
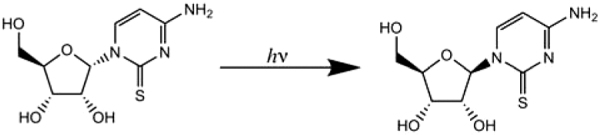


The synthesis of ribonucleotides from 2-aminooxazole and glyceraldehyde followed by reaction with cyanoacetylene and phosphorylation results in β-ribocytidine, one of the building blocks of RNA (Powner *et al.*, 2007, 2009). However, this synthesis utilized a minor product of the reaction with 2-aminooxazole and glyceraldehyde, arabinose aminooxazoline (AAO, 30% yield), ignoring the major product (isomer), ribose aminooxazoline (RAO, 44% yield) (Sanchez and Orgel, 1970), which selectively crystallizes from the product solution, and allows enantiomeric enrichment during crystallization if the input glyceraldehyde is nonracemic (Anastasi *et al.*, 2006). Reaction of RAO with cyanoacetylene followed by hydrolysis results in α-ribocytidine, which is a stereoisomer of the RNA building block β-ribocytidine. α-Ribocytidine can be photochemically converted into β-ribocytidine, but the conversion is inefficient, due to the competing formation of a by-product, oxazolidinone (Powner *et al.*, 2007).

Recently, Xu *et al.* (2018) optimized the synthesis of canonical pyrimidine ribonucleosides/tides (genetic alphabet C and U), starting with RAO. In this synthesis, α-anhydrocytidine which results from RAO, reacts with hydrogen sulfide to produce α-thiocytidine, which is efficiently photoanomerized into β-thiocytidine. β-Thiocytidine either reacts slowly with water or phosphate to produce β-ribocytidine (C) and β-ribouridine (U). The above Eq. 8 is the critical step converting α-thiocytidine to β-thiocytidine.

α-Thiocytidine (0.015 mmol, 4.0 mg) and NaSH.xH_2_O (0.021 mmol, 2 mg, containing >60% of NaSH) were mixed in argon-degassed H_2_O (5 mL, 10% D_2_O/90% H_2_O). The pH of the mixture was adjusted to 7.0 with 1 *M* HCl. The mixture was then divided into two 10 mm quartz cuvettes, one of which was irradiated at 254 nm in a photochemical chamber reactor RPR-200, while the other sample was irradiated in StarLab for the same time.

As can be seen from the NMR spectra, shown in Fig. 9, the photoanomerization in StarLab after 7 h of irradiation results in a 50% yield of β-thiocytidine, and a 90% yield after 15 h of irradiation. This corresponds to a timescale of 360–600 h for 50% yield on the surface of early Earth. If the lifetime of the relevant intermediates is on the order of this timescale, then this pathway will be plausible. The timescale can also be compared with that of the Rayonet, which achieves 50% yield in half the time, but the yield does not improve past 50%. This suggests that the less intense and more realistic broadband source can drive the reaction while suppressing or changing other reactions that destroy the product.

### 3.5. Attempted photoreduction of thioanhydrouridine

The fourth reaction we consider is as follows:

**9:**
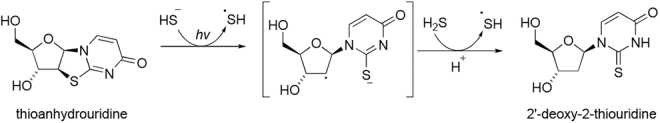


Many believe that the first informational macromolecule on early Earth was the RNA (*e.g*., Gilbert, 1986), for reasons involving the capabilities of chemical synthesis (Noorden, 2009), and evidence that certain RNA molecules are capable both of storing information and catalyzing reactions, both in the laboratory (Orgel, 2004) and in modern organisms (Cech, 2000). Finally, it would seem challenging for a system involving eight monomers, four ribonucleotides, and four deoxyribonucleotides to self-assemble into informational molecules that self-replicate with sufficient fidelity (Eigen, 1971), in the absence of macromolecular machines to chaperone, segregate, and facilitate both ribonucleotide and deoxyribonucleotide chemistries. It is worth noting that prebiotic chemists, such as Orgel (1968), were open to the possibility of DNA preceding RNA, or of both arising simultaneously.

Recently, Bhowmik and Krishnamurthy (2019) have postulated and provided some proof of concept that hybrid RNA-DNA systems can segregate themselves into homogeneous RNA and DNA macromolecules. They discovered that certain chimeric templates catalyze the ligation of oligomers, resulting in homogeneous RNA and DNA sequences.

An interesting development in prebiotic nucleoside synthesis is the recent report of the photoreduction of an RNA thionucleoside derivative, thioanhydrouridine, to a DNA thionucleoside, 2′-deoxy-2-thiouridine (Xu *et al.*, 2019). The discovery provides an explanation for the origin of the building blocks of DNA on early Earth alongside those of RNA, much earlier than usually thought. The reaction was proposed to proceed via photodetachment of an electron from HS^−^, which reduces the C2′–S bond of the thionucleoside, followed by protonation and hydrogen atom transfer. Photodetachment was affected by the RPR-200 with 254 nm lamps, leading to the production of 2′-deoxy-2-thiouridine in 33% yield after 3 h.

We performed identical experiments in the RPR-200 with 254 nm lamps and in StarLab, to verify the plausibility of this process on early Earth. We found the result reproducible in the RPR-200, and no reaction to occur in StarLab, even with extended radiation times up to 15 h.

To a solution of 2,2′-anhydro-2-thiouridine (0.066 mmol, 16 mg, 1.0 equiv.) in argon-degassed water (2.40 mL, 10% D_2_O/90% H_2_O) was added a solution of NaSH.xH_2_O (60% NaSH; 70 mg, 0.38 mmol, 11 equiv.) dissolved in 2.0 mL degassed water (10% D_2_O/90% H_2_O). The pH of the mixture was adjusted to 7.0 by using degassed 1 *M* HCl. The mixture was then divided into two 10 mm quartz cuvettes, each containing ∼2.2 mL with a sample height of 22 mm. One sample was irradiated at 254 nm in a Rayonet photochemical chamber reactor RPR-200 for 3 h. The other sample was irradiated in StarLab for the same time.

After 3 h, the reaction in the Rayonet reactor showed a similar outcome to that reported in the work of Xu *et al.* (2019), with a mixture of reduction product 2S-dU, starting material, and thiouracil being detected (24%, 64%, and 12%, respectively). The reactions in StarLab showed only recovered starting material.

Similar experiments were conducted with longer irradiation times in StarLab of 5 and 15 h, with some of the sample being simultaneously submitted to irradiation in the Rayonet reactor for 3 h. In each case, photoreduction proceeded in the Rayonet and did not in the StarLab. Unreacted samples recovered from the StarLab trials were submitted to irradiation in the Rayonet reactor and showed some conversion. Since the photoreduction did not proceed, we cannot make any robust estimates about timescales for plausibility. We can only provide an upper limit of the timescale for this reaction of 1000 h, assuming a detection limit of 1% yield. This implies a lower limit for the timescale of this reaction on the surface of early Earth of several years.

### 3.6. Photoreduction of thioanhydroadenosine

A related photoreduction to the reaction discussed in the previous section is as follows:

**10:**
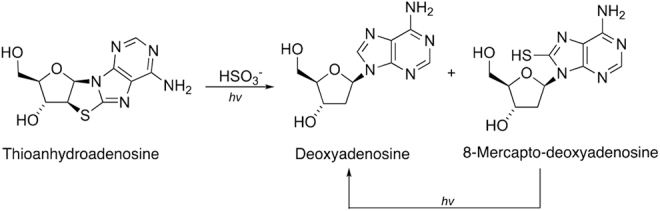


Soon after the synthesis worked out by Xu *et al.* (2019, 2020), discovered a prebiotic pathway from 8-mercaptoadenine and α-anhydropyrimidines to form thioanhydroadenosine, which can then be photoreduced in the presence of bisulfite to form 2′-deoxyriboadenosine (dA). The final step requires UV light, and bears some similarity to the failed reaction with hydrogen sulfide to form 2′-deoxyribopyrimidines, above, and so this is the reaction we test with StarLab.

Thioanhydroadenosine (0.014 mmol, 4.0 mg) and Na_2_SO_3_ (0.143 mmol, 18.0 mg) were mixed in argon-degassed H_2_O (4.0 mL, 10% D_2_O/90% H_2_O). The pH of the mixture was adjusted to 7.0 with degassed HCl (1.0 *M*). The mixture was then divided into two 10 mm quartz cuvettes, one of which was irradiated at 254 nm in a photochemical chamber reactor RPR-200, while the other sample was irradiated in StarLab for the same time.

**FIG. 10.**
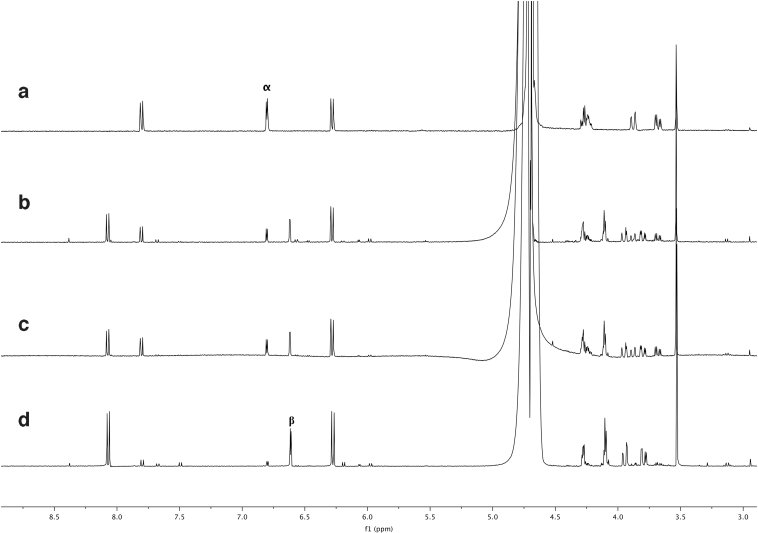
Photoanomerization of α-thiocytidine. One can compare the height of the peak at ∼6.8 ppm (reactant, labeled α) and the peak at ∼6.6 ppm (product, labeled β). As can be seen, StarLab achieves a better overall yield than the Rayonet (RPR-200) but is slower. **(a)** ^1^H NMR spectrum of the mixture in the dark; **(b)** ^1^H NMR spectrum of the mixture after 7 h of irradiation in the RPR-200 reactor. **(c)** ^1^H NMR spectrum of the mixture after 7 h of irradiation in StarLab. **(d)** ^1^H NMR spectrum of the mixture after 15 h of irradiation in StarLab. The rates are comparable between the RPR-200 and StarLab, and after 15 h, irradiation with StarLab results in a higher yield than RPR-200. These results imply a half-life for photoanomerization with StarLab of 7 h.

After 2 h of irradiation in RPR-200, 60% of reduced products (including 2′-deoxyadenosine and 8-mercapto-2′-deoxyadenosine) were observed in crude NMR spectra, while the conversion in StarLab was 40%. After another 2 h of irradiation, the yield of identifiable reduced products had decreased to 40%, possibly due to decomposition by the 185 nm spectral feature produced by the low-pressure Hg lamp. After the same amount of time, the yield of reduced products in StarLab had increased slightly to 43%. The results are shown in Fig. 10.

Although in this case, the kinetics does not simply allow for a simple application of Eq. 6, this equation applies an upper limit to the timescale of 180–300 h for similar chemical starting conditions to result in a yield of ∼40% on the surface of early Earth, a much shorter timescale than for the DNA pyrimidines.

### 3.7. Nitroprusside

We now consider the following reaction:

**11:**
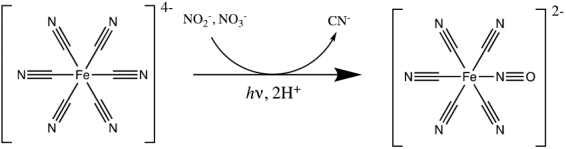


The synthesis of life's building blocks is only the very beginning of the origins of life problem (Sutherland, 2017). To construct biological macromolecules from these building blocks, the synthesized molecules need to undergo oligomerization or ligation at the correct positions, typically by being activated with a chemical reagent. In modern cells, this is accomplished with enzymes, but enzymes in modern cells are synthesized by those cells. There is no clear abiotic synthetic pathway for enzymes, and so, it seems that there must be a mechanism for nonenzymatic activation of life's building blocks.

One promising means of activating ribonucleotides, amino acids, and phospholipids involves methyl isocyanide (Bonfio *et al.*, 2019; Liu *et al.*, 2020), and one prebiotically plausible synthesis of methyl isocyanide begins with ferrocyanide and nitrites or nitrates (Mariani *et al.*, 2018b). The immediate reaction these undergo in the presence of UV light is the formation of nitroprusside, and this is the reaction we consider here.

Several possible pathways lead from ferrocyanide ([Fe^II^(CN)_6_]^4−^) to nitroprusside [Fe^II^(CN)_5_NO]^2−^ (Mariani *et al.*, 2018b). Oxidation of ferrocyanide to ferricyanide ([Fe^III^(CN)_6_]^3−^) by NO_2_^−^ yields one equivalent of nitric oxide (NO^**•**^) and one equivalent of H_2_O. Subsequently, photodissociation of one cyanide ligand from ferricyanide gives [Fe^III^(CN)_5_]^2−^, a coordinatively unsaturated intermediate in equilibrium with the aqua complex [Fe^III^(CN)_5_H_2_O]^2−^, which then equilibrates with NO^**•**^ to form nitroprusside **(**[Fe^II^(CN)_5_NO]^2−^). Alternatively, since photolysis of NO_2_^−^ provides a hydroxyl radical (OH^**•**^) in addition to NO**^•^** (Mack and Bolton, 1999), the oxidation of ferro- to ferricyanide may be mediated by the hydroxyl radical. Clearly the production of nitroprusside from ferrocyanide involves a complex multistep mechanism and so analyzing the kinetics is more involved.

First, we investigated the formation of [Fe^II^(CN)_5_NO]^2−^ upon irradiation of a mixture of [Fe^II^(CN)_6_]^4−^ and NO_2_^−^, as described previously (Mariani *et al.*, 2018b). Second, as nitrate (NO_3_^−^) would likely have been more abundant than NO_2_^−^ on early Earth (Ranjan *et al.*, 2019), and since photolysis of NO_3_^−^ also provides NO**^•^** and OH^**•**^ (Mack and Bolton, 1999), we investigated the formation of [Fe^II^(CN)_5_NO]^2−^ upon irradiation of mixtures of [Fe^II^(CN)_6_]^4−^ and NO_3_^−^, or both NO_2_^−^ and NO_3_^−^.

Experiments were performed as follows: Na_4_[Fe^II^(CN)_6_]·10H_2_O (0.1 mmol, 48.4 mg) and NaNO_2_ (0.1 mmol, 6.9 mg), NaNO_3_ (0.1 mmol, 8.5 mg), or a mixture of NaNO_2_ (0.05 mmol, 3.5 mg) and NaNO_3_ (0.05 mmol, 4.3 mg) were dissolved in 1.0 mL of an argon-degassed 0.1 *M* solution of imidazole in water (10% D_2_O/90% H_2_O). The pH of the mixture was adjusted to 7.0 by using deoxygenated HCl (1.0 *M*). The mixture was then transferred to a 10 mm quartz cuvette and irradiated in either the RPR-200, with 254 nm lamps or 365 nm lamps, or in StarLab for 1 h. [Fe^II^(CN)_5_NO]^2−^ was detected by ^13^C NMR spectroscopy (number of scans: 1000; recycle delay time: 2 s; NOESY mixing time: 0 s; TOCSY mixing time: 0 s; ROESY mixing time: 0 s).

**FIG. 11.**
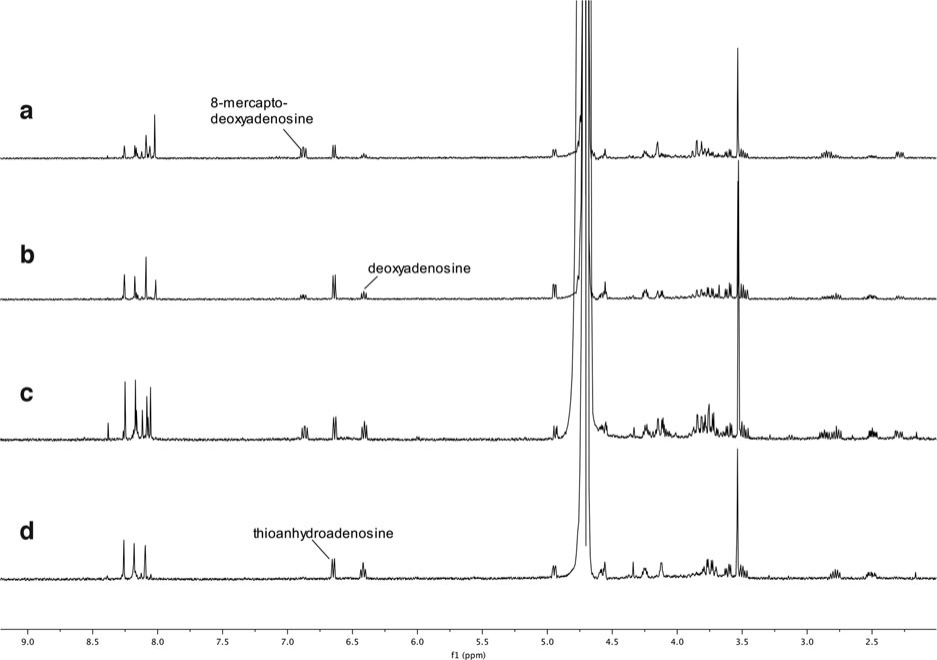
NMR spectra, namely **(a)** crude ^1^H-NMR spectrum after irradiation in Rayonet (RPR-200) for 2 h; **(b)** crude ^1^H-NMR spectrum after irradiation in StarLab for 2 h; **(c)** crude ^1^H-NMR spectrum after irradiation in Rayonet (RPR-200) for 4 h; **(d)** crude ^1^H-NMR spectrum after irradiation in StarLab for 4 h. As with the photoanomerization reaction (Fig. 10), the yield is better for StarLab, but the reaction is much slower.

**FIG. 12.**
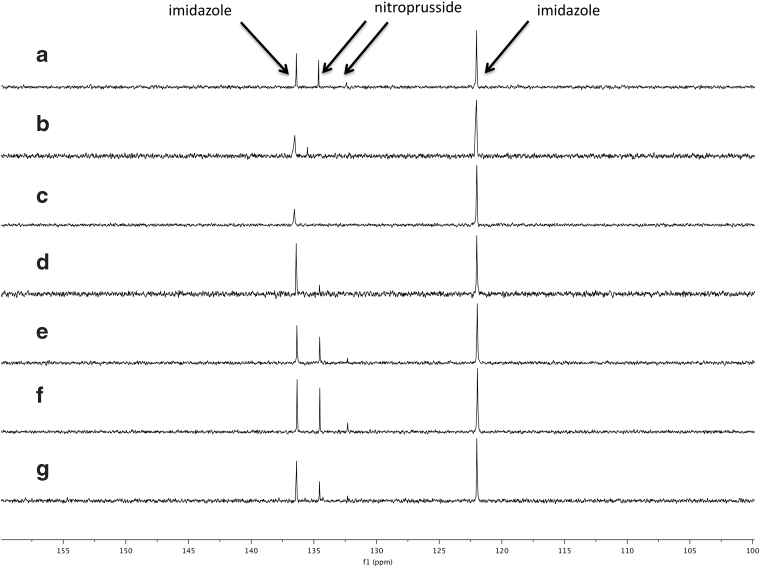
^13^C NMR of ferrocyanide mixed with NO_2_^−^/NO_3_^−^ and irradiated, as follows. **(a)** Reaction in StarLab using a mixture of NO_2_^−^ and NO_3_^−^. **(b)** Reaction in StarLab using NO_3_^−^. **(c)** Reaction in Rayonet (365 nm) using NO_3_^−^. **(d)** Reaction in Rayonet (254 nm) using NO_3_^−^. **(e)** Reaction in StarLab using NO_2_^−^. **(f)** Reaction in Rayonet (365 nm) using NO_2_^−^. **(g)** Reaction in Rayonet (254 nm) using NO_2_^−^. The area under the curve for nitroprusside product can be compared with the imidazole (which acts as a standard), and the NMR ratios can be compared with nitroprusside standards, as shown in Fig. 13. NO_3_^−^, nitrate.

To quantify the amount of [Fe^II^(CN)_5_NO]^2−^ formed in each experiment, a standard curve was made by measuring ^13^C NMR spectra (parameters as described above) of different concentrations of Na_2_[Fe^II^(CN)_5_NO] in an imidazole buffer (0.1 *M*) at pH 7.0. Each measurement was made three times. A plot of the integral of the [Fe^II^(CN)_5_NO]^2−^ signal at *δ* 134.5 ppm relative to the integral of the imidazole signal at *δ* 122.0 ppm, versus the concentration of [Fe^II^(CN)_5_NO]^2−^, shows a linear correlation. The ^13^C NMR spectra of the reactions described above are shown in Fig. 11 and the linear regression of NMR signal ratios as a function of known quantities of nitroprusside, compared with experimental data, is shown in Fig. 12.

**FIG. 13.**
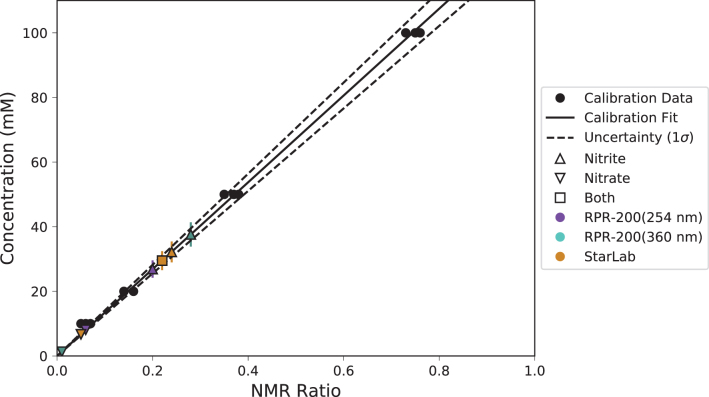
Known concentrations of nitroprusside (m*M*) as a function of the ^13^C NMR peak integral ratio of nitroprusside to imidazole (see Section 3.7 for details). These clearly follow a linear relationship, and a line is fit to the data with 1boldσbold uncertainties shown. The same ratios were calculated for the ferrocyanide irradiation data (Fig. 12), and the concentrations are estimated with 1boldσbold errors. Color images are available online.

We found that irradiation of a mixture of [Fe^II^(CN)_6_]^4−^ and NO_2_^−^ in an imidazole (0.1 *M*) buffer at pH 7.0 with the RPR-200, using either 254 or 365 nm lamps, or light from StarLab for 1 h gave [Fe^II^(CN)_5_NO]^2−^ in concentrations of 26.9 ± 2.7 m*M* for the RPR-200 (254 nm), 37.6 ± 3.8 m*M* for the RPR-200 (365 nm), and 32.2 ± 3.2 m*M* for StarLab. These concentrations correspond to yields of 27% for the RPR-200 (254 nm), 38% for the RPR-200 (365 nm), and 33% for StarLab.

Irradiation of a mixture of [Fe^II^(CN)_6_]^4−^ and NO_3_^−^ in an imidazole buffer (0.1 *M*) at pH 7.0 with light of 254 nm or light from StarLab for 1 h gave [Fe^II^(CN)_5_NO]^2−^ in concentrations of 8.1 ± 0.8 m*M* for the RPR-200 (254 nm), ≤1.3 m*M* for the RPR-200 (365 nm) and 6.7 ± 0.7 m*M* for StarLab, corresponding to yields of ∼10% in the cases of the RPR-200 (254 nm) and StarLab, and ∼2% for the RPR-200 (365 nm). Irradiation of this mixture with light of 365 nm provided no detectable [Fe^II^(CN)_5_NO]^2−^, hence the upper limit. Finally, irradiation of a mixture of [Fe^II^(CN)_6_]^4−^, NO_2_^−^, and NO_3_^−^ in an imidazole buffer (0.1 *M*) at pH 7.0 with light from StarLab for 1 h also gave [Fe^II^(CN)_5_NO]^2−^ in a concentration of 29.5 ± 2.9 m*M*, corresponding to a yield of about 30%.

As before, we can use the yields to estimate the timescales of these reactions on the surface of early Earth. For the reaction of ferrocyanide with NO_2_^−^ or with NO_2_^−^ and NO_3_^−^, it would take 36–60 h to achieve a 50% yield of nitroprusside. With only NO_3_^−^, the timescale is on the order of a month. If ferrocyanide is stable on an anoxic early Earth on these timescales, then nitroprusside is prebiotically plausible in pools of water rich in NO_2_^−^, NO_3_^−^, or both.

### 3.8. Hydroxyl radical production—EtOH

Photochemistry is not only constructive. Photodetachment of electrons from anions can occur with OH^−^ anions ever present in liquid water. The product is a solvated electron and a hydroxyl radical, and hydroxyl radicals can be devastating to life's building blocks and prebiotic intermediate species. The cross section for photodetachment of hydride fortuitously peaks below 200 nm (Zuman and Szafranski, 1976) and both CO_2_ and H_2_O vapor effectively shield the surface from these wavelengths (Ranjan and Sasselov, 2016), and so, any planet with a substantial (>0.1 bar) atmosphere will be protected from these wavelengths.

We first found with the Rayonet RPR-200 reactor that photodetachment of an electron from OH^−^ may occur above 200 nm. There is then the potential that hydroxyl radicals would be photochemically produced on early Earth but not in some photoreactors typically used in the laboratory, although hydroxyl radicals may be overproduced in other laboratory reactors, such as low-pressure mercury lamps by their 184 nm emission feature. We test this possibility with StarLab. We explore the oxidation of EtOH by hydroxyl radicals in this context.

We mix EtOH (0.18 mmol, 10.5 μL) in argon-degassed water (6 mL, 10% D_2_O/90% H_2_O). The solution has a pH of 7.5. The mixture is divided into three cuvettes, one irradiated by StarLab for 2 h, one irradiated by the RPR-200 for 2 h, and a third that is not irradiated. The third cuvette is a control in the event that the argon degassing is not sufficient to prevent some oxidation of EtOH. We dissolved pentaerythritol (25 μmol, 3.4 mg) in argon-degassed H_2_O (0.5 mL) resulting in a 50 m*M* solution. After irradiation, we added the pentaerythritol solution (166 μL) to each of the three samples as a standard to estimate the concentrations of the oxidation products.

The ^1^H NMR results show no detectable irradiation products of EtOH, such as acetate, acetaldehyde, and 1,1-ethanediol for either the control cuvette or the StarLab-irradiated cuvette. The RPR-200-irradiated cuvette shows some photooxidation products, likely due to the 184 nm feature (Fig. 13).

**FIG. 14.**
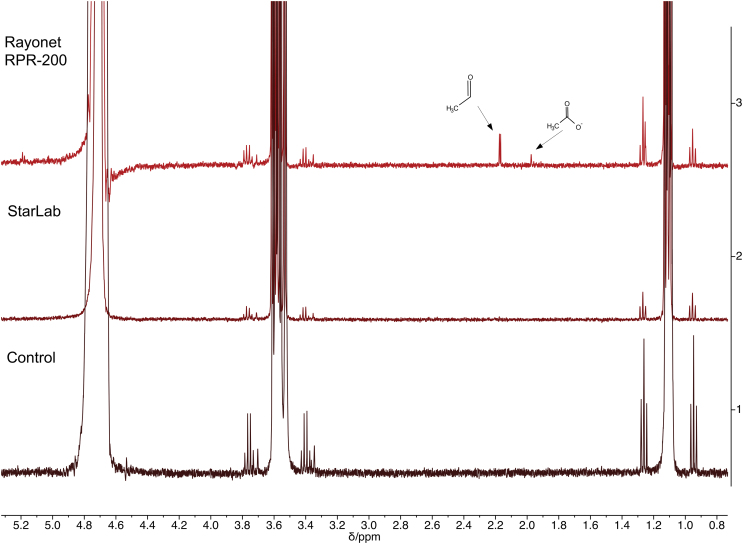
Irradiation of ethanol for 2 h by the Rayonet RPR-200 (top), StarLab (middle), and left in the dark (bottom, control). No observable oxidation products after 2 h for either StarLab or the Control. Some oxidation products were visible with the Rayonet. Color images are available online.

**FIG. 15.**
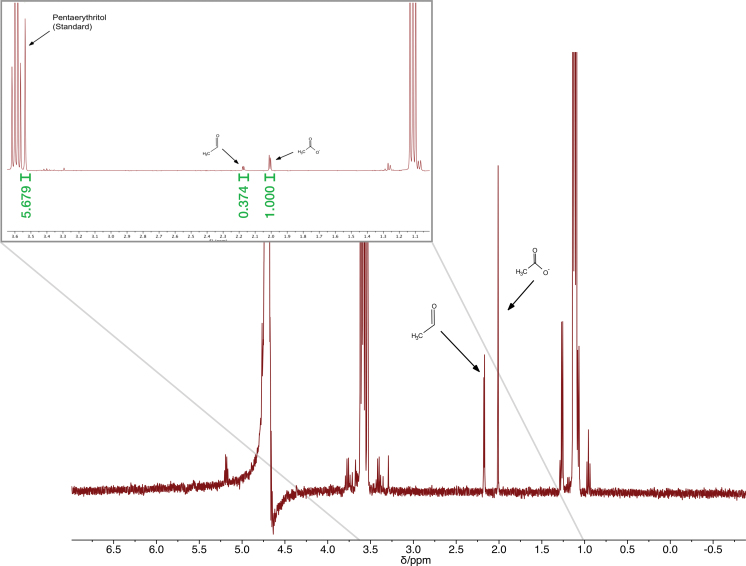
Irradiation of ethanol by StarLab for 24 h. Now some products are detected and their line integrals can be compared with the line integral for pentaerythritol to estimate the concentrations of the oxidation products. Color images are available online.

We repeated the experiment, but with EtOH (0.36 mmol, 21 μL), and we irradiated the mixture with StarLab for 24 h (Figs. 14 and 15). Some oxidation products were observed. By integrating under the spectral curves for the oxidation products and for pentaerythritol, we can estimate the concentration of the oxidation products.

Taking the total integral of the oxidation products to be 1.88, when accounting for the ^1^H degeneracies and duplicate peaks, and the total integral of pentaerythritol is 0.71, after accounting for the ^1^H degeneracies for pentaerythritol (degeneracy of 8). The concentration of the oxidation products is then as follows:

(12) $$c_{ox}=\frac{c_{0}I_{ox}}{I_{0}}$$

where $c_{ox}eqnoopen\left(M\right)$ is the concentration of the oxidation products, $I_{ox}$ is the integral of the lines for the oxidation species, adjusting for degeneracies and duplicate peaks, $c_{0}eqnoopen\left(M\right)$ is the concentration of the pentaerythritol, and *I*_0_ the integral of the pentaerythritol feature, accounting for degeneracies.

We find that *c*_0_ = 0.002 *M*, *I*_0_ = 0.71, and $I_{ox}$ = 1.88, so $c_{ox}$ = 0.005 *M*. Since the starting concentration of EtOH was 0.06 *M*, this is a yield of 8%. We apply this yield to Eq. 6 to estimate the time it would take to achieve a yield of 50% of 290 h with StarLab, or more than a year under the light of the young Sun.

## 4. Discussion

Using the results from the beginning of Section 3, we can draw out a relationship between the StarLab spectral irradiance and the UV actinic flux on the surface of early Earth. That relationship is wavelength dependent, because the spectrum of the young Sun is not identical to the StarLab spectrum, even considered at low resolution. Also, the atmosphere and the local aqueous chemistry can affect the UV environment. We made the assumption that the atmosphere is composed of 0.3 bar N_2_/CO/CO_2_ with H_2_O at vapor pressure, which is mostly transparent to UV light above 200 nm.

We also assume an aqueous environment, as may be found in a mineral-rich postimpact crater pond with ferrocyanide at concentrations of >0.1 m*M*, which at shallow depths of a few mm if rich in ferrocyanide blocks UV light below 220 nm (Higashi and Ozaki, 2004; Chakrabarti and Roberts, 2008). This environment is approximated with the use of mineral water (Cambridge tap water) within the quartz attenuator into which the experimental samples are placed. To be clear, the tap water only attenuates the UV light and is not in contact with the samples themselves.

**FIG. 16.**
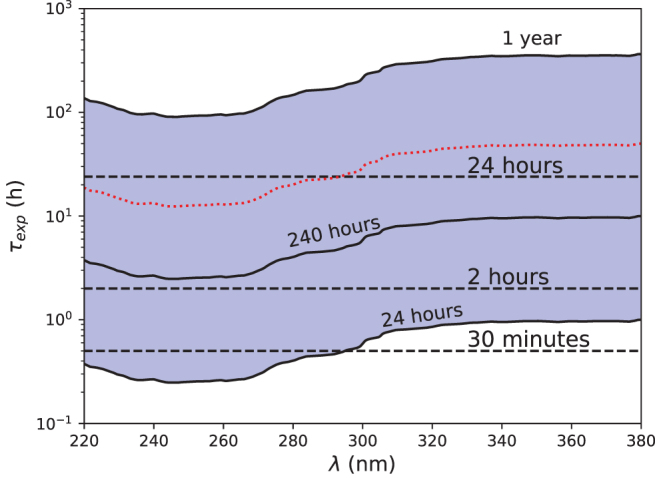
A comparison between the time an experiment should reach 50% yield in StarLab (h), to reach a similar yield on the surface of early Earth (with atmosphere as described in Fig. 5) as a function of wavelength (nm). Dashed lines show experimental reaction times measured for StarLab, and solid lines the equivalent reaction time on the surface of early Earth. The dotted red line is the degradation timescale for bisulfite and HCN (from Rimmer *et al*., 2018). Color images are available online.

We show the relationship between the experimental timescales and timescales for reactions on the surface of early Earth as a function of wavelength in Fig. 16. Our results agree reasonably well with those of Ranjan and Sasselov (2016).

Of course, the atmosphere of early Earth when life arose may have been different than we assume. It may have been more reducing, and if so, there would likely have been thick photochemical hazes that can obscure the UV light between 200 and 300 nm (Arney *et al.*, 2016). If life's origins were coincident with the tail end of accretion, hazes generated by accretionary impact may obscure the UV light (Rimmer *et al.*, 2020). NH_3_ would also attenuate UV light below 230 nm (Cheng *et al.*, 2006). The results in this article provide timescales under optimal conditions for surface prebiotic photochemistry.

The surface UV flux predicted by Rimmer *et al.* (2018) does not match the surface fluxes predicted by Ranjan *et al.* (2017), and this is due to our assignment of *a* < 1 single-scattering albedo when calculating Rayleigh scattering. The result has been to effectively include a small particulate haze and overattenuate the short-wavelength NUV compared with a clear sky model. Using the surface UV flux predicted by Rimmer *et al.* (2018) with single-scattering albedo set to unity, to better approximate clear skies, we find any reaction that achieves a yield of 50% after a 12-h exposure to StarLab meets the abiogenesis zone requirements of Rimmer *et al.* (2018) for the young Sun. Future work will investigate the implications of these experimental results for abiogenesis zones around other stars. The timescale related to the abiogenesis zone of Rimmer *et al.* (2018) is shown in Fig. 16.

This abiogenesis zone affords a “ballpark” estimate for degradation timescales, based on a chemist's experience with these reagents in the laboratory, many of the “stable” intermediates tend to have lifetimes comparable with HCN and bisulfite, although lifetimes for these species depend on temperature and pH and can range over many orders of magnitude (see, *e.g*., Miyakawa *et al.*, 2002).

To genuinely test whether these reactions would work on early Earth or around another planet, the degradation chemistry needs to be explored over a range of conditions, and applied to the analysis, to determine whether, in the context of a particular scenario, a given reaction is likely to occur with reasonable yield on early Earth, on the basis of the available UV light. Nevertheless, constraints on plausibility can be made based on the known stability of the reactants in the laboratory. Reactants that cannot survive more than a few days must achieve high yields in a matter of a few hours under StarLab, or the degradation will win out, and the path is not prebiotically plausible.

We explore the prebiotic plausibility of six different reactions (Sections 3.2–3.8), and summarize our results in Table 1. We find that most of the reactions, the photooxidation of hypophosphite, the photochemical reaction of HCN and bisulfite, the photoanomerization of thiocytidine, and the conversion of ferrocyanide to nitroprusside, all appear plausible from the standpoint of UV light, although the timescale for photoanomerization of thiocytidine (approximately 360–600 h on early Earth) suggests that the yield for this reaction may be suboptimal, and that further exploration into alternative mechanisms for producing β-ribocytidine is warranted.

**Table 1.** Timescales for Key Prebiotic Photochemical Reactions on the Surface of Early Earth, Accounting for the Light of the Young Sun

Reaction	Time irradiated in the laboratory (h)	Yield in the laboratory	Irradiation timescale^a^	Early Earth timescale^b^
Oxidation of hypophosphite	1	50%	18–30 h	36–60 h
HCN+bisulfite	1	10%	90–150 h	180–300 h
Photoanomerization of α-thiocytidine	7	50%	180–300 h	360–600 h
Photoreduction of thioanhydrouridine	15	<1%	>5 Years	>10 Years
Photoreduction of thioanhydroadenosine	2	40%	90–150 h	180–300 h
Nitroprusside synthesis	1	30% with nitrite and nitrite+nitrate, 10% with nitrate	18–30 h with nitrite, ∼720 h without nitrate	36–60 h with nitrite, ∼1440 h without nitrate
Oxidation of ethanol	24	8%	>1 Year	>2 Years

^a^ The time the sample must be irradiated on the surface of early Earth to obtain a yield of 50%.

^b^ The time the sample must be present on early Earth to obtain a yield of 50%, accounting for the day/night cycle.

HCN = hydrogen cyanide.

In particular, we found that nitroprusside is plausible in water with NO_3_^−^, even if no NO_2_^−^ is present, and that the photodestruction of nitroprusside by light at short wavelengths is outcompeted by the photochemical production of nitroprusside, likely owing to the spectral shape of StarLab and of the young Sun, producing much more light at longer wavelengths (*i.e*., >254 nm). This effect will be further enhanced if short wavelength light is attenuated by the water, as was the case for <220 nm with StarLab. This attenuation may have other consequences for the rest of the network, and opens up the possibility for chemical temporal segregation within the same body of water, where one region of water experiences a different chemical environment. If there is inefficient mixing, or if there are ways to segregate different parts of a pool (pores or vesicles), chemical heterogeneity and relevant chemical gradients will be generated within a pond simply by UV attenuation. Further investigation into this possibility is needed.

We finally turn to the photoreduction of thioanhydrouridine with hydrogen sulfide, photoreduction of thioanhydroadenosine with bisulfite, and the production of hydroxyl radicals from water, quantified via the subsequent oxidation of EtOH. The photoreduction of thioanhydrouridine and production of hydroxyl radicals from water are not plausible. In the case of oxidation of EtOH, this implies that the hydroxyl production by the young Sun on early Earth is very slow, and should not interfere with the prebiotic chemistry.

The failure to photoreduce thioanhydrouridine allowed us to place a lower limit on the timescale for this photochemistry of several years. Even this lower limit may be optimistic, however, considering that it assumes the reaction would occur given longer irradiation times. This may be implausible because the presence of light at other wavelengths may have opened up alternative interfering pathways, or may have altogether disrupted the mechanism allowed under the Rayonet RPR-200.

Finally, the related photoreduction of thioanhydroadenosine, a similar nucleoside derivative, was successful when using bisulfite as the source of solvated electrons. This reaction worked rapidly in StarLab, achieving the final ∼40% yield in 2 h. This suggests that bisulfite is a more plausible source of solvated electrons than hydrogen sulfide, and that more than one step in the prebiotic scenario we consider requires the presence of bisulfite in order for the UV light to be prebiotic.

Future investigations of the prebiotic plausibility of this scenario should focus on the contingency of various reactions in sequence, with a demonstration that these reactions can run in sequence, either without the aid of the experimentalist or with that aid explained by a plausible physical process.

The intervention required to complete the chemistry can be used to estimate the probability that this chemistry could have taken place on early Earth, early Mars, or on other rocky planets outside our solar system. In addition, careful investigation into chemical mechanisms, such as the work done when using pump-probe techniques from the works of Roberts *et al.* (2018) and Todd *et al.* (2019), will be key for understanding *why* some reactions behave differently under a broadband source than under a single wavelength source. This understanding will also be essential for discovering alternative pathways once certain pathways are falsified in terms of the available UV light.

### 4.1. Other effects of stellar irradiation and activity

Stellar irradiation and activity have other effects on planetary atmospheres and the surface environment (for a review of stellar activity through time, see Güdel 2007). Although UV light at wavelengths <200 nm cannot reach the surface of a planet with *a* > 0.1 bar CO_2_/N_2_ atmosphere, this light drives atmospheric escape (discussed in Gronoff *et al.*, 2020), and can erode atmospheres and can transform them. If the atmosphere is fully eroded, the planet will not be habitable.

Stellar activity can also result in the abiotic buildup of molecular oxygen (Tian *et al.*, 2014), which would interfere with several prebiotic chemical reactions, including the ones discussed here, which generally require anoxic conditions, and any scenario that is inhibited or disrupted by molecular oxygen, for example, chemistry that makes use of cysteine-containing peptides (Bonfio *et al.*, 2017). Energetic photons are not all bad. They can lead to the formation of HCN, although in relatively small quantities (Zahnle, 1986; Tian *et al.*, 2011; Rimmer and Rugheimer, 2019), and of formaldehyde (Pinto *et al.*, 1980), precursors for the UV-driven prebiotic scenario we investigate here.

Energetic particles can penetrate deeper into the atmosphere and may cause more severe problems for atmospheric evolution. Energetic particles, produced from coronal mass ejections that accompany flares, are more relevant for surface chemistry, since their chemical impact lies within the stratosphere and troposphere, rather than being relegated to the thermosphere and mesosphere (Airapetian *et al.*, 2016). These solar energetic particles generate greenhouse gases, which may affect the surface temperature of early Earth and generate substantial amounts of HCN (Airapetian *et al.*, 2016).

Solar energetic particles can themselves drive some prebiotic chemistry in the gas phase (Kobayashi *et al.*, 1998). The light of flares may be relevant for the >200 nm UV environment on the surface of rocky exoplanets (see Section 4.2), but are not relevant for early Earth. We can see this by comparing the energy deposited by the light of the Sun with the energy deposited by a high-energy flare that the active cool star AD Leo produced (Hawley and Pettersen, 1991). This flare has energy >10 × the energy of the Carrington event (Cliver and Dietrich, 2013), and is thought to be at the upper limit of flare energies for the Sun (Cliver and Dietrich, 2013). As can be seen from the work of Segura *et al.* (2010), the intensity of this flare only achieves the quiescent emission of the young Sun.

### 4.2. Implication for exoplanets

One great advantage of this scenario is that it depends on starlight, and starlight is the property of exoplanet systems that is best known. As discussed above, the measurement of the rates of these reactions under realistic conditions in the laboratory, and the comparison of these rates with degradation rates for the reactants, allows us to investigate whether these reactions are likely to take place on the surface of planets around other stars. To investigate this chemistry in the context of exoplanets, changes to the StarLab configuration, as presented in this article, will be implemented.

To investigate the chemistry that may be enabled by including the 200–220 nm UV wavelengths in our current broad wavelength range, we use ultrapure water in the quartz attenuator. As already discussed above, most plausible aqueous environments on early Earth would not include these shorter wavelengths, but to investigate chemistry that could happen on the actual surface layer, this band of the UV would be available, and these microenvironments should also be explored in the future.

Looking beyond the environment on early Earth, we also plan to simulate the stellar flux environment as may be experienced on exoplanet surfaces around other stellar types to experimentally constrain the abiogenesis zone based not on one reaction, but based on the chemistry as a whole, using a realistic light source that accurately represents the diverse spectra of these stars.

In addition, we look into the effects of catalysts on the UV-driven chemistry, for example, the catalytic role of ferrocyanide in the cyanide reduction by UV-irradiation of bisulfite. This is especially interesting for M dwarf stars—due to their smaller size and mass, Earth-like, rocky planets are more easily detected compared with Earth-like planets around solar-like stars.

A number of Earth-like exoplanets around M dwarfs have already been discovered, such as the TRAPPIST-1 system (Gillon *et al.*, 2017) and planets similar to these will be good candidates for atmospheric characterization with the James-Webb telescope. It is therefore interesting to investigate if UV-driven origin chemistry on such planets is plausible—on these very red M stars sufficient UV flux only comes from flares. In a future article we will present a reconfiguration of StarLab to simulate the flux environment (including periodic flares), as may be experienced on planets around M stars, and determine if any of the chemical pathways presented here are possible in such a nonconstant UV flux environment, and to provide an update to the abiogenesis zone based on stellar activity.

## 5. Conclusions

We constructed a stellar simulator that accurately reproduces a multiple of the broadband actinic flux of the young Sun on the surface of early Earth. We then performed a series of seven experiments with StarLab exploring the various stages of a prebiotic chemical scenario with UV-driven aqueous chemistry starting with cyanide, bisulfite, and sulfides, with a focus on the role of the UV light. We first considered the availability of phosphate, an essential feedstock molecule for subsequent prebiotic synthesis, by exploring the photooxidation of hypophosphite with hydrogen sulfide. Then we explored the first steps of the synthesis, which commences with HCN and bisulfite. Next, we turned to a key later synthetic step toward cytidine and uridine, the photoanomerization of thiocytidine.

Then we attempted the photoreduction of thioanhydrouridine, a key step in the prebiotic synthesis of deoxynucleotides. This photoreduction failed in StarLab, allowing us to place a lower limit on the timescale for this reaction on early Earth, assuming longer exposures would result in the expected product. In Section 4, we present some reasons why this is unlikely to be the case, and that it is more likely new destructive chemical mechanisms have been initiated due to the broadband nature of the light and the nonlinear nature of the chemistry. We also demonstrated the successful photoreduction of thioanhydroadenosine in the presence of bisulfite, indicating that bisulfite is a necessary condition for the sufficiency of solar UV light for prebiotic photochemistry.

Next, we considered the production of nitroprusside, the first step in the prebiotically plausible production of methyl isocyanide. Methyl isocyanide can be used as a component in the nonenzymatic activation of nucleotides, amino acids, and phospholipid precursors. Finally, we considered destructive hydroxyl radical production by exploring the oxidation of EtOH by UV-generated hydroxyl radicals in water.

We discovered that the photooxidation of hypophosphite, the reaction of HCN with bisulfite, and the production of nitroprusside are all prebiotically plausible, and the photoanomerization of thiocytidine is at the edge of plausibility. We additionally discovered that nitroprusside can be produced from ferrocyanide and NO_3_^−^ alone, and that the rate of this conversion suggests that nitroprusside is prebiotically plausible in both NO_2_^−^-rich and NO_3_^−^-rich water, so long as the water also has access to ferrocyanide and UV light.

We found that two reactions, photoreduction of thioanhydrouridine and oxidation of EtOH, are not prebiotically plausible. In the former case, there are alternative pathways to RNA pyrimidines and DNA purines that do succeed, although with the use of bisulfite instead of hydrogen sulfide as the source of free electrons. In the latter case, this is a positive outcome for the prebiotic chemistry, because the results of the EtOH oxidation experiment suggest that timescales for producing hydroxyl radicals in water on the surface of early Earth are likely far longer than the lifetimes of even stable intermediates. However, if certain building blocks need to survive for more than a matter of decades, then the production of hydroxyl radicals may become significant.

One advantage of exploring prebiotic plausibility in terms of starlight is that starlight is what exoplanet scientists observe first and know best. Starlight is also one of the best constrained global parameters for early Earth, although even here uncertainty remains (Spalding *et al.*, 2018). Constraining prebiotic plausibility by using starlight will apply to all the local environments on early Earth, and the constraints can be made with a higher level of confidence than those for other parameters such as chemical composition or local temperature or pH.

These constraints can also be transformed into conditions for further delineating the abiogenesis zone for the Sun, and for other stars, including for ultracool stars. This is especially relevant given our present knowledge about the UV environments of planetary systems around ultracool host stars such as TRAPPIST-1 (Ducrot *et al.*, 2020) and systems discovered by TESS (Günther *et al.*, 2020), and future discoveries around these systems, as well as around young exoplanet systems (Bottrill *et al.*, 2020; Rimmer *et al.*, 2020). If biosignatures are discovered on a statistically significant subset of these planets, the distribution of that subset may provide the only real way to test prebiotic chemical scenarios in the future.

## Abbreviations Used

EtOH: ethanol
HCN: hydrogen cyanide
NH_3_: ammonia
NO: nitric oxide
NO_2_: nitrogen dioxide
NO_2_^−^: nitrite
NO_3_^−^: nitrate
OH^−^: hydroxide
PH_3_: phosphine
RAO: ribose aminooxazoline
UV: ultraviolet

## Notes

Associate Editor: Nita Sahai

## References

[B1] Airapetian VS, Glocer A, Gronoff G, *et al.* (2016) Prebiotic chemistry and atmospheric warming of early Earth by an active young Sun. Nat Geosci 9:452–455.

[B2] Anastasi C, Crowe MA, Powner MW, *et al.* (2006) Direct assembly of nucleoside precursors from two- and three-carbon units. Angew Chem Int Ed Engl 45:6176–6179. 16917794 10.1002/anie.200601267

[B3] Arney G, Domagal-Goldman SD, Meadows VS, *et al.* (2016) The pale orange dot: the spectrum and habitability of hazy Archean Earth. Astrobiology 16:873–899. 27792417 10.1089/ast.2015.1422 PMC5148108

[B4] Avice G, Marty B, and Burgess R (2017) The origin and degassing history of the Earth's atmosphere revealed by Archean xenon. Nat Commun 8:15455. 28516958 10.1038/ncomms15455 PMC5454381

[B5] Bhowmik S and Krishnamurthy R (2019) The role of sugar-backbone heterogeneity and chimeras in the simultaneous emergence of RNA and DNA. Nat Chem 11:1009–1018. 31527850 10.1038/s41557-019-0322-x PMC6815252

[B6] Bonanno A, Schlattl H, and Paternò L (2002) The age of the Sun and the relativistic corrections in the EOS. Astron Astrophys 390:1115–1118.

[B7] Bonfio C, Valer L, Scintilla S, *et al.* (2017) UV-light-driven prebiotic synthesis of iron–sulfur clusters. Nat Chem 9:1229–1234. 29168482 10.1038/nchem.2817 PMC5808832

[B8] Bonfio C, Caumes C, Duffy CD, *et al.* (2019) Length-selective synthesis of acylglycerol-phosphates through energy-dissipative cycling. J Am Chem Soc 141:3934–3939. 30767518 10.1021/jacs.8b12331 PMC6506141

[B9] Bottrill AL, Haigh ME, Hole MRA, *et al.* (2020) Exoplanet detection and its dependence on stochastic sampling of the stellar Initial Mass Function. Astrophys J 895:141.

[B10] Brasser R, Werner SC, and Mojzsis SJ (2020) Impact bombardment chronology of the terrestrial planets from 4.5 Ga to 3.5 Ga. Icarus 338:113514.

[B11] Bryant DE, Greenfield D, Walshaw RD, *et al.* (2013) Hydrothermal modification of the Sikhote-Alin iron meteorite under low pH geothermal environments. A plausibly prebiotic route to activated phosphorus on the early Earth. Geochim Cosmochim Acta 109:90–112.

[B12] Burkholder JB, Sander SP, Abbatt JPD, *et al.* (2020) Chemical kinetics and photochemical data for use in atmospheric studies; evaluation number 19. Jet Propulsion Laboratory, National Aeronautics and Space Administration, Pasadena, CA.

[B13] Canavelli P, Islam S, and Powner MW (2019) Peptide ligation by chemoselective aminonitrile coupling in water. Nature 571:546–549. 31292542 10.1038/s41586-019-1371-4

[B14] Cech TR (2000) The ribosome is a ribozyme. Science 289:878–879. 10960319 10.1126/science.289.5481.878

[B15] Chakrabarti MH and Roberts EPL (2008) Analysis of mixtures of ferrocyanide and ferricyanide using UV-visible spectroscopy. J Chem Soc Pak 30:817.

[B16] Chen Y and Zhu L (2001) The wavelength dependence of the photodissociation of propionaldehyde in the 280–330nm region. J Phys Chem 105:9689–9696.

[B17] Cheng B, Lu H, Chen H, *et al.* (2006) Absorption cross sections of NH_3_, NH2D, NHD2, and ND3 in the spectral range 140–220nm and implications for planetary isotopic fractionation. Astrophys J 647:1535.

[B18] Claire MW, Sheets J, Cohen M, *et al.* (2012) The evolution of solar flux from 0.1nm to 160μm: quantitative estimates for planetary studies. Astrophys J 757:95.

[B19] Cliver EW and Dietrich WF (2013) The 1859 space weather event revisited: limits of extreme activity. J Space Weather Space Clim 3:A31.

[B20] Cousins CR, Crawford IA, Carrivick JL, *et al.* (2013) Glaciovolcanic hydrothermal environments in Iceland and implications for their detection on Mars. J Volcanol Geotherm Res 256:61–77.

[B21] Ducrot E, Gillon M, Delrez L, *et al.* (2020) TRAPPIST-1: global results of the Spitzer Exploration Science Program Red Worlds. Astron Astrophys 640:A112.

[B22] Eigen M (1971) Self-organization of matter and the evolution of biological macromolecules. Naturwissenschaften 58:465–523. 4942363 10.1007/BF00623322

[B23] Gaillard F and Scaillet B (2014) A theoretical framework for volcanic degassing chemistry in a comparative planetology perspective and implications for planetary atmospheres. Earth Planet Sci Lett 403:307–316.

[B24] Genda H, Brasser R, Mojzsis SJ (2017) The terrestrial late veneer from core disruption of a lunar-sized impactor. Earth Planet Sci Lett 480:25–32.

[B25] Gilbert W (1986) Origin of life: the RNA world. Nature 319:618.

[B26] Gillon M, Triaud AHMJ, Demory B-O, *et al.* (2017) Seven temperate terrestrial planets around the nearby ultracool dwarf star TRAPPIST-1. Nature 542:456–460. 28230125 10.1038/nature21360 PMC5330437

[B27] Glindemann D, Edwards M, and Kuschk P (2003) Phosphine gas in the upper troposphere. Atmos Environ 37:2429–2433.

[B28] Gronoff G, Arras P, Baraka S, *et al.* (2020) Atmospheric escape processes and planetary atmospheric evolution. J Geophys Res Space Phys 125:e2019JA027639. DOI: 10.1029/2019JA027639.

[B29] Güdel M (2007) The Sun in time: activity and environment. Living Rev Sol Phys 4:3.

[B30] Gulick A (1955) Phosphorus as a factor in the origin of life. Am Sci 43:479–489.

[B31] Günther MN, Zhan Z, Seager S, *et al.* (2020) Stellar flares from the first TESS data release: exploring a new sample of M dwarfs. Astron J 159:60. DOI: 10.3847/1538-3881/ab5d3a.

[B32] Hawley SL and Pettersen BR (1991) The great flare of 1985 April 12 on AD Leonis. Astrophys J 378:725.

[B33] Higashi N and Ozaki Y (2004) Potential of far-ultraviolet absorption spectroscopy as a highly sensitive quantitative and qualitative analysis method for aqueous solutions, Part I: determination of hydrogen chloride in aqueous solutions. Appl Spectrosc 58:910–916. 15324496 10.1366/0003702041655331

[B34] Huebner WF, Keady JJ, and Lyon SP (1992) Solar photo rates for planetary atmospheres and atmospheric pollutants. Astrophys Space Sci 195:1–294.

[B35] Huebner WF and Mukherjee J (2015) Photoionization and photodissociation rates in solar and blackbody radiation fields. Planet Space Sci 106:11–45.

[B36] Ityaksov D, Linnartz H, and Ubachs W (2008) Deep-UV absorption and Rayleigh scattering of carbon dioxide. Chem Phys Lett 462:31–34.

[B37] Kadoya S, Krissansen-Totton J, and Catling DC (2020) Probable cold and alkaline surface environment of the Hadean Earth caused by impact ejecta weathering. Geochem Geophys Geosyst 21:e2019GC008734. DOI: 10.1029/2019GC008734.

[B38] Kee T, Bryant D, Herschy B, *et al.* (2013) Phosphate activation via reduced oxidation state phosphorus (P). Mild routes to condensed-P energy currency molecules. Life 3:386–402. 25369812 10.3390/life3030386 PMC4187178

[B39] Keller-Rudek H, Moortgat GK, Sander R, *et al.* (2013) The MPI-Mainz UV/VIS spectral atlas of gaseous molecules of atmospheric interest. Earth Syst Sci Data 5:365–373.

[B40] Kim Y, Demarque P, Yi SK, *et al.* (2002) The Y^2^ isochrones for α-element enhanced mixtures. Astrophys J Suppl Ser 143:499.

[B41] Kobayashi K, Kaneko T, Saito T, *et al.* (1998) Amino acid formation in gas mixtures by high energy particle irradiation. Orig Life Evol Biosph 28:155–165. 11536862 10.1023/a:1006561217063

[B42] Krissansen-Totton J, Arney GN, and Catling DC (2018) Constraining the climate and ocean pH of the early Earth with a geological carbon cycle model. Proc Natl Acad Sci U S A 115:4105–4110. 29610313 10.1073/pnas.1721296115 PMC5910859

[B43] Lehmer OR, Catling DC, Buick R, *et al.* (2020) Atmospheric CO_2_ levels from 2.7 billion years ago inferred from micrometeorite oxidation. Sci Adv 6:eaay4644. DOI: 10.1126/sciadv.aay4644. 32010786 PMC6976288

[B44] Liu Z, Wu LF, Xu J, *et al.* (2020) Harnessing chemical energy for the activation and joining of prebiotic building blocks. Nat Chem 12:1023–1028. 33093680 10.1038/s41557-020-00564-3 PMC7610406

[B45] Luo G, Ono S, Beukes NJ, *et al.* (2016) Rapid oxygenation of Earth's atmosphere 2.33 billion years ago. Sci Adv 2:e1600134. 27386544 10.1126/sciadv.1600134 PMC4928975

[B46] Mack J and Bolton JR (1999) Photochemistry of nitrite and nitrate in aqueous solution: a review. J Photochem Photobiol Chem 128:1–13.

[B47] Mariani A, Bonfio C, Johnson CM, *et al.* (2018a) pH-Driven RNA strand separation under prebiotically plausible conditions. Biochemistry 57:6382–6386. 30383375 10.1021/acs.biochem.8b01080 PMC6340128

[B48] Mariani A, Russell DA, Javelle T, *et al.* (2018b) A light-releasable potentially prebiotic nucleotide activating agent. J Am Chem Soc 140:8657–8661. 29965757 10.1021/jacs.8b05189 PMC6152610

[B49] Mittag M, Schröder K-P, Hempelmann A, *et al.* (2016) Chromospheric activity and evolutionary age of the Sun and four solar twins. Astron Astrophys 591:A89. DOI: 10.1051/0004-6361/201527542.

[B50] Miyakawa S, Cleaves HJ, and Miller SL (2002) The cold origin of life: B. Implications based on pyrimidines and purines produced from frozen ammonium cyanide solutions. Orig Life Evol Biosph 32:209–218. 12227425 10.1023/a:1019514022822

[B51] Nash WP (1984) Phosphate minerals in terrestrial igneous and metamorphic rocks. In Phosphate Minerals, edited by J.O. Nriagu and P.H. Moore, Springer, Berlin, Heidelberg, p 215.

[B52] Noorden RV (2009) RNA world easier to make. Nature 459:239–242.19444213

[B53] Orgel LE (1968) Evolution of the genetic apparatus. J Mol Biol 38:381–393. 5718557 10.1016/0022-2836(68)90393-8

[B54] Orgel LE (2004) Prebiotic chemistry and the origin of the RNA world. Crit Rev Biochem Mol Biol 39:99–123. 15217990 10.1080/10409230490460765

[B55] Patel BH, Percivalle C, Ritson DJ, *et al.* (2015) Common origins of RNA, protein and lipid precursors in a cyanosulfidic protometabolism. Nat Chem 7:301–307. 25803468 10.1038/nchem.2202 PMC4568310

[B56] Payne RC, Brownlee D, and Kasting JF (2020) Oxidized micrometeorites suggest either high pCO2 or low pN2 during the Neoarchean. Proc Natl Acad Sci U S A 117:1360–1366. 31907311 10.1073/pnas.1910698117 PMC6983370

[B57] Pinto JP, Gladstone GR, and Yung YL (1980) Photochemical production of formaldehyde in Earth's primitive atmosphere. Science 210:183–185. 17741284 10.1126/science.210.4466.183

[B58] Powner MW, Anastasi C, Crowe MA, *et al.* (2007) On the prebiotic synthesis of ribonucleotides: photoanomerisation of cytosine nucleosides and nucleotides revisited. Chembiochem 8:1170–1179. 17549787 10.1002/cbic.200700098

[B59] Powner MW, Gerland B, and Sutherland JD (2009) Synthesis of activated pyrimidine ribonucleotides in prebiotically plausible conditions. Nature 459:239–242. 19444213 10.1038/nature08013

[B60] Quickenden TI and Irvin JA (1980) The ultraviolet absorption spectrum of liquid water. J Chem Phys 72:4416.

[B61] Ranjan S and Sasselov DD (2016) Influence of the UV environment on the synthesis of prebiotic molecules. Astrobiology 16:68–88. 26789356 10.1089/ast.2015.1359

[B62] Ranjan S, Todd ZR, Sutherland JD, *et al.* (2018) Sulfidic anion concentrations on early earth for surficial origins-of-life chemistry. Astrobiology 18:1023–1040. 29627997 10.1089/ast.2017.1770 PMC6225604

[B63] Ranjan S, Todd ZR, Rimmer PB, *et al.* (2019) Nitrogen oxide concentrations in natural waters on early Earth. Geochem Geophys Geosyst 20:2021–2039.

[B64] Ranjan S, Schwieterman EW, Harman C, *et al.* (2020) Photochemistry of anoxic abiotic habitable planet atmospheres: impact of new H_2_O cross sections. Astrophys J 896:148.

[B65] Ribas I, Guinan EF, Gudel M, *et al.* (2005) Evolution of the solar activity over time and effects on planetary atmospheres. I. High-energy irradiances (1-1700 Å). Astrophys J 622:680.

[B66] Ribas I, Porto de Mello GF, Ferreira LD, *et al.* (2010) Evolution of the solar activity over time and effects on planetary atmospheres. II. κ1 Ceti, an analog of the Sun when life arose on Earth. Astrophys J 714:384.

[B67] Rimmer PB and Helling C (2016) A chemical kinetics network for lightning and life in planetary atmospheres. ApJS 224:9.

[B68] Rimmer PB and Rugheimer S (2019) Hydrogen cyanide in nitrogen-rich atmospheres of rocky exoplanets. Icarus 329:124–131.

[B69] Rimmer PB and Shorttle O (2019) Origin of life's building blocks in carbon-and nitrogen-rich surface hydrothermal vents. Life (Basel) 9:12. 10.3390/life9010012 PMC6463091 30682803

[B70] Rimmer PB, Xu J, Thompson SJ, *et al.* (2018) The origin of RNA precursors on exoplanets. Sci Adv 4:eaar3302. DOI: 10.1126/sciadv.aar3302. PMC6070314 30083602

[B71] Rimmer PB, Shorttle O, and Rugheimer S (2019) Oxidised micrometeorites as evidence for low atmospheric pressure on the early Earth. Geochem Perspect Lett 9:38–42. 31187073 10.7185/geochemlet.1903 PMC6558283

[B72] Rimmer PB, Ferus M, Waldmann IP, *et al.* (2020) Identifiable acetylene features predicted for young Earth-like exoplanets with reducing atmospheres undergoing heavy bombardment. Astrophys J 888:21.

[B73] Ritson DJ and Sutherland JD (2012) Prebiotic synthesis of simple sugars by photoredox systems chemistry. Nat Chem 4:895–899. 23089863 10.1038/nchem.1467 PMC3589744

[B74] Ritson DJ, Mojzsis SJ, and Sutherland JD (2020) Supply of phosphate to early Earth by photogeochemistry after meteoritic weathering. Nat Geosci 13:344–348. 32395178 10.1038/s41561-020-0556-7 PMC7213494

[B75] Roberts SJ, Szabla R, Todd ZR, *et al.* (2018) Selective prebiotic conversion of pyrimidine and purine anhydronucleosides into Watson-Crick base-pairing arabino-furanosyl nucleosides in water. Nat Commun 9:1–10. DOI: 10.1038/s41467-018-06374-z. 30287815 10.1038/s41467-018-06374-z PMC6172253

[B76] Sagan C and Mullen G (1972) Earth and Mars: evolution of atmospheres and surface temperatures. Science 177:52–56. 17756316 10.1126/science.177.4043.52

[B77] Sanchez RA and Orgel LE (1970) Studies in prebiotic synthesis: V. Synthesis and photoanomerization of pyrimidine nucleosides. J Mol Biol 47:531–543. 5418170 10.1016/0022-2836(70)90320-7

[B78] Sasselov DD, Grotzinger JP, and Sutherland JD (2020) The origin of life as a planetary phenomenon. Sci Adv 6:eaax3419. DOI: 10.1126/sciadv.aax3419. 32076638 PMC7002131

[B79] Segura A, Walkowicz LM, Meadows V, *et al.* (2010) The effect of a strong stellar flare on the atmospheric chemistry of an Earth-like planet orbiting an M dwarf. Astrobiology 10:751–771. 20879863 10.1089/ast.2009.0376 PMC3103837

[B80] Som SM, Buick R, Hagadorn JW, *et al.* (2016) Earth's air pressure 2.7 billion years ago constrained to less than half of modern levels. Nat Geosci 9:448–451.

[B81] Spalding C, Fischer WW, and Laughlin G (2018) An orbital window into the ancient Sun's mass. Astrophys J 869:L19. DOI: 10.3847/2041-8213/aaf219.

[B82] Stairs S, Nikmal A, Bučar D-K, *et al.* (2017) Divergent prebiotic synthesis of pyrimidine and 8-oxo-purine ribonucleotides. Nat Commun 8:1–12. DOI: 10.1038/ncomms15270. 28524845 10.1038/ncomms15270 PMC5454461

[B83] Sutherland JD (2015) The origin of life—out of the blue. Angew Chem Int Ed Engl 55:104–121. 26510485 10.1002/anie.201506585

[B84] Sutherland JD (2017) Opinion: studies on the origin of life—the end of the beginning. Nat Rev Chem 1:1–7. DOI: 10.1038/s41570-016-0012.

[B85] Tackett SL, Meyer WM, Pany FG, *et al.* (1966) Electrolytic dissolution of iron meteorites. Science 153:877–880. 17780649 10.1126/science.153.3738.877

[B86] Tian F, Kasting JF, and Zahnle K (2011) Revisiting HCN formation in Earth's early atmosphere. Earth Planet Sci Lett 308:417–423.

[B87] Tian F, France K, Linsky JL, *et al.* (2014) High stellar FUV/NUV ratio and oxygen contents in the atmospheres of potentially habitable planets. Earth Planet Sci Lett 385:22–27.

[B88] Todd ZR, Fahrenbach AC, Magnani CJ, *et al.* (2018) Solvated-electron production using cyanocuprates is compatible with the UV-environment on a Hadean–Archaean Earth. Chem Commun Camb Engl 54:1121–1124. 10.1039/c7cc07748c PMC9631354 29334083

[B89] Todd ZR, Szabla R, Szostak JW, *et al.* (2019) UV photostability of three 2-aminoazoles with key roles in prebiotic chemistry on the early earth. Chem Commun 55:10388–10391. 10.1039/c9cc05265h PMC9631353 31380533

[B90] Tomkins AG, Bowlt L, Genge M, *et al.* (2016) Ancient micrometeorites suggestive of an oxygen-rich Archaean upper atmosphere. Nature 533:235–238. 27172047 10.1038/nature17678

[B91] Toner JD and Catling DC (2019) A carbonate-rich lake solution to the phosphate problem of the origin of life. Proc Natl Acad Sci U S A 117:883–888. 31888981 10.1073/pnas.1916109117 PMC6969521

[B92] Wu LF and Sutherland JD (2019) Provisioning the origin and early evolution of life. Emerg Top Life Sci 3:459–468. 32002470 10.1042/ETLS20190011 PMC6992421

[B93] Xu J, Ritson DJ, Ranjan S, *et al.* (2018) Photochemical reductive homologation of hydrogen cyanide using sulfite and ferrocyanide. Chem Commun Camb Engl 54:5566–5569. 10.1039/c8cc01499j PMC5972737 29761807

[B94] Xu J, Green NJ, Gibard C, *et al.* (2019) Prebiotic phosphorylation of 2-thiouridine provides either nucleotides or DNA building blocks via photoreduction. Nat Chem 11:457–462. 30936523 10.1038/s41557-019-0225-x PMC6597365

[B95] Xu J, Chmela V, Green NJ, *et al.* (2020) Selective prebiotic formation of RNA pyrimidine and DNA purine nucleosides. Nature 582:60–66. 32494078 10.1038/s41586-020-2330-9 PMC7116818

[B96] Yi SK, Kim Y-C, and Demarque P (2003) The Y2 stellar evolutionary tracks. Astrophys J Suppl Ser 144:259–261.

[B97] Zahnle KJ (1986) Photochemistry of methane and the formation of hydrocyanic acid (HCN) in the Earth's early atmosphere. J Geophys Res Atmos 91:2819–2834.

[B98] Zahnle KJ, Lupu R, Catling DC, *et al.* (2020) Creation and evolution of impact-generated reduced atmospheres of early Earth. Planet Sci J 1:11. DOI: 10.3847/PSJ/ab7e2c.

[B99] Zuman P and Szafranski W (1976) Ultraviolet spectra of hydroxide, alkoxide, and hydrogen sulfide anions. Anal Chem 48:2162–2163.

